# High-resolution X-ray diffraction determination of the electron density of 1-(8-PhSC_10_H_6_)SS(C_10_H_6_SPh-8′)-1′ with the QTAIM approach: evidence for S_4_ σ(4c–6e) at the naphthalene *peri*-positions†

**DOI:** 10.1039/c7ra13636f

**Published:** 2018-03-05

**Authors:** Yutaka Tsubomoto, Satoko Hayashi, Waro Nakanishi, Lucy K. Mapp, Simon J. Coles

**Affiliations:** Faculty of Systems Engineering, Wakayama University 930 Sakaedani Wakayama 640-8510 Japan nakanisi@sys.wakayama-u.ac.jp hayashi3@sys.wakayama-u.ac.jp +81 73 457 8253 +81 73 457 8252; Chemistry, Faculty of Natural and Environmental Sciences, University of Southampton Southampton SO17 1BJ UK S.J.Coles@soton.ac.uk +44 (0)2380596721

## Abstract

An extended hypervalent S_4_ σ(4c–6e) system was confirmed for the linear ^B^S-∗-^A^S-∗-^A^S-∗-^B^S interaction in 1-(8-Ph^B^SC_10_H_6_)^A^S–^A^S(C_10_H_6_^B^SPh-8′)-1′ (1) *via* high-resolution X-ray diffraction determination of electron densities. The presence of bond critical points (BCPs; ∗) on the bond paths confirms the nature and extent of this interaction. The recently developed QTAIM dual functional analysis (QTAIM-DFA) approach was also applied to elucidate the nature of the interaction. Total electron energy densities *H*_b_(***r***_c_) were plotted *versus H*_b_(***r***_c_) − *V*_b_(***r***_c_)/2 for the interaction at the BCPs, where *V*_b_(***r***_c_) represents the potential energy densities at the BCP. The results indicate that although the data for an interaction in the fully optimized structure corresponds to a static nature, the data obtained for the perturbed structures around it represent the dynamic nature of the interaction in QTAIM-DFA. The former classifies the interaction and the latter characterises it. Although ^A^S-∗-^A^S in 1 is classified by a shared shell interaction and exhibits weak covalent character, ^A^S-∗-^B^S is characterized as having typical hydrogen-bond nature with covalent properties in the region of the regular closed shell interactions. The experimental results are supported by matching theoretical calculations throughout, particularly for the extended hypervalent E_4_ σ(4c–6e) (E = S) interaction.

## Abstract 2

The nature of S_4_ σ(4c–6e) at the 1,8-positions of naphthalene is elucidated *via* a high-resolution X-ray method with the QTAIM approach.

## Graphical abstract

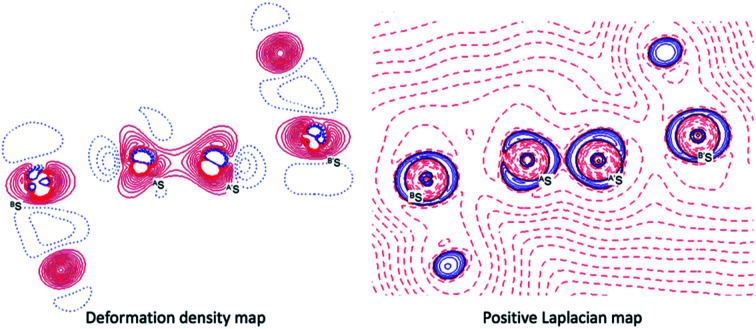

## Introduction

**Scheme 1:**
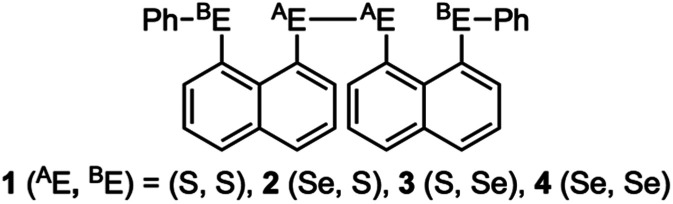
Structures of 1–4.

Recent research in our laboratory has been concerned with linear σ-type interactions higher than σ(3c–4e), which is the classical three-center four-electron σ type bonds/interactions.^1^ We refer to such interactions as extended hypervalent σ(*m*c–*n*e) (4 ≤ *m*; *m* < *n* < 2*m*).^2–7^ The σ(4c–6e) interaction is the first to be studied. ^A^E_2_^B^E_2_ σ(4c–6e) was first recognized for the linear alignments of four ^B^E⋯^A^E–^A^E⋯^B^E atoms in the structures of bis[8-(phenylchalcogena)naphthyl]-1,1′-dichalcogenide [1-(8-Ph^B^EC_10_H_6_)^A^E–^A^E(C_10_H_6_^B^EPh-8′)-1′ (^A^E, ^B^E) = (S, S: 1),^3^ (S, Se: 2),^4^ (Se, S: 3)^4^ and (Se, Se: 4)^2^] as determined by X-ray crystallographic analysis (see Fig. 2 for the structure of 1 (S, S) determined by the high-resolution X-ray crystallographic analysis). Scheme 1 illustrates the structures of 1–4. A substantial number of compounds containing the σ(4c–6e) interaction have been reported.^6,8,9^ Benzene substituents in the 1,2-positions, naphthalene substituents in the 1,8-positions and related systems serve as good spacers for the formation of these interactions.^2–4,10–12^ It has been strongly suggested that σ(4c–6e) interactions play an important role in the development of high functionalities in materials and also in key processes of biological and/or pharmaceutical activities.^2–4,6–12^

**Fig. 1:**
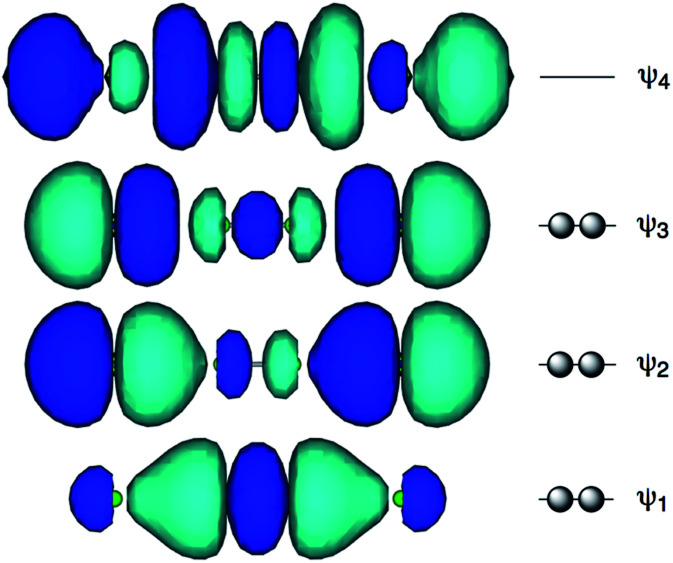
Approximate MO model for E_4_ σ(4c–6e) exemplified by Cl_4_^2−^ (E = Cl).

Fig. 1 shows the molecular orbitals of E_4_ σ(4c–6e), which is exemplified in the case of Cl_4_^2−^ (E = Cl). The central ^A^E–^A^E distance in ^B^E⋯^A^E–^A^E⋯^B^E should be shorter than the ^A^E⋯^B^E distances in σ(4c–6e) even if ^A^E = ^B^E. This expectation is supported by the optimized structure of E_4_^2−^ (E = Cl) (Fig. 1). Charge transfer (CT) of the n_p_(^B^E)→σ∗(^A^E–^A^E)←n_p_(^B^E) type is the driving force for the formation of ^A^E_2_^B^E_2_ σ(4c–6e), where n_p_(^B^E) represents the p-type lone pair orbitals of ^B^E with σ∗(^A^E–^A^E) for the σ∗ orbital of ^A^E–^A^E. The high accepting ability of σ∗(^A^E–^A^E) and the high donating ability of n_p_(^B^E) plays an important role in the formation of the linear interaction. The nature of ^A^E_2_^B^E_2_ σ(4c–6e) is well established theoretically. The dynamic and static nature of ^A^E_2_^B^E_2_ σ(4c–6e) is elucidated for 1-(8-Me^B^EC_10_H_6_)^A^E–^A^E(C_10_H_6_^B^EMe-8′)-1′: (^A^E, ^B^E) = (S, S: 5), (S, Se: 6), (Se, S: 7) and (Se, Se: 8), after preparation and structural determination of 8.^13^ The static nature of ^A^E_2_^B^E_2_ σ(4c–6e) has also been reported for 1–4 based on the observed crystal structures.^13–16^^A^E_2_^B^E_2_ σ(4c–6e) are also recognized as a form of chalcogen bonding.^17^

**Fig. 2:**
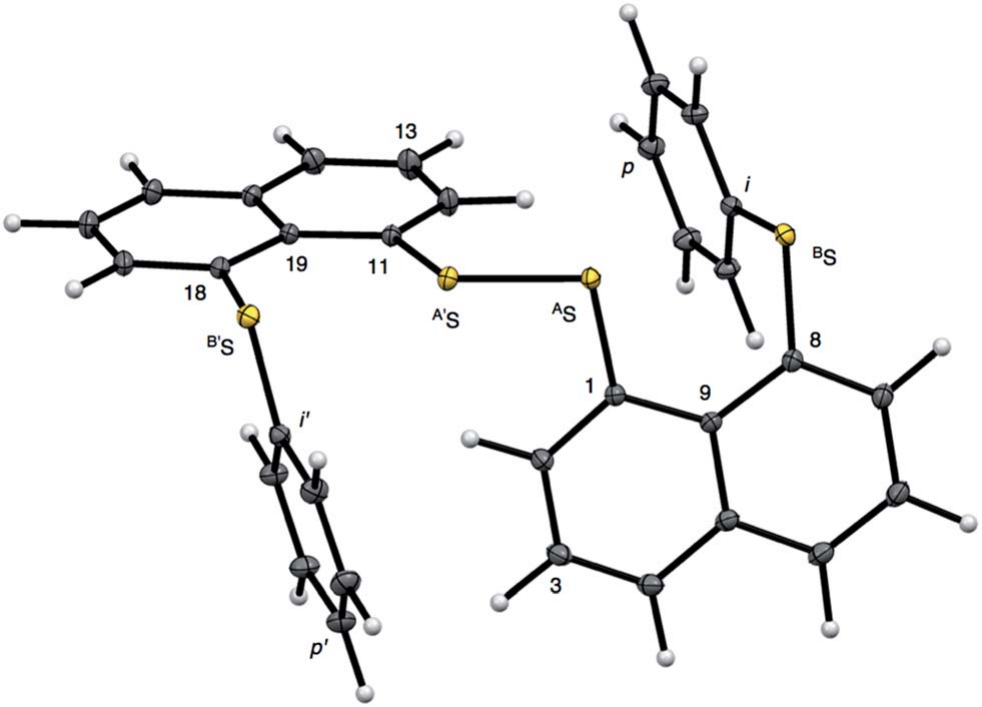
Structure of 1 (S, S) determined by high-resolution X-ray crystallographic analysis.

Thus, the question of how the nature of ^A^E_2_^B^E_2_ σ(4c–6e) can be established experimentally arises. The high-resolution X-ray diffraction determination of electron densities of 1 (S, S) would provide a firm basis for the real existence of S_4_ σ(4c–6e) in 1 (S, S). The quantum theory of atoms-in-molecules dual functional analysis (QTAIM-DFA), which we proposed recently,^18–22^ will support the experimental results by elucidating the dynamic and static nature of S_4_ σ(4c–6e) from the observed and/or optimized structures of 1 (S, S). It will be easily understood if the interactions can be defined by the corresponding bond paths (BPs) in QTAIM, but we must carefully use the correct terminology with the concept.^23^ A basis set system that reproduces the observed structural parameters, particularly for the ^A^E⋯^B^E distances, *r*(^A^E⋯^B^E), must be established.

Therefore, this paper is concerned with the observation of the existence of S_4_ σ(4c–6e) in 1 (S, S) based on the data obtained from high-resolution X-ray diffraction determination of electron densities. The real existence of S_4_ σ(4c–6e) in 1 (S, S) was confirmed by theoretically elucidating the nature of S_4_ σ(4c–6e) with QTAIM-DFA. The nature of the interactions was determined by employing the criteria proposed previously when applying QTAIM-DFA to typical chemical bonds and interactions. QTAIM-DFA and the criteria are explained in the ESI in Scheme S1 and S2, Fig. S1 and eqn (S1)–(S6).† The basic concept of the QTAIM approach introduced by Bader^24–26^ is also surveyed, which enables us to analyze the nature of chemical bonds and interactions.^27–33^

## Experimental

### Bis[8-(phenylthio)naphthyl]-1,1′-disulfide (1 (S, S))

Compound 1 (S, S) was obtained *via* the reaction of the naphtho[1,8-*c*,*d*]-1,2-dithiolate dianion^3,4^ with excess benzenediazonium chloride in aqueous THF at 2–4 °C. After standard workup, the solution was chromatographed on silica gel containing acidic alumina. Recrystallization from the solvent mixed with hexane and dichloromethane afforded 1 (S, S) as yellow prisms in 68% yield, mp 169.8–170.6 °C. ^1^H NMR (CDCl_3_/TMS, 400 MHz) *δ* 7.02 (dd, *J* = 1.2 and 8.3 Hz, 4H), 7.12 (t, *J* = 7.7 Hz, 4H), 7.21 (t, *J* = 7.3 Hz, 4H), 7.45 (t, *J* = 7.6 Hz, 2H), 7.63 (dd, *J* = 1.0 and 8.1 Hz, 2H), 7.68 (dd, *J* = 1.1 and 7.6 Hz, 2H), 7.85 (dd, *J* = 1.4 and 7.2 Hz, 2H), 7.90 (dd, *J* = 1.3 and 8.2 Hz, 2H); ^13^C NMR (CDCl_3_/TMS, 75.5 MHz) *δ* 125.5, 125.8, 125.8, 126.2, 127.1, 127.4, 128.4, 128.9, 131.4, 133.8, 134.5, 136.4, 138.5 and 139.9 (see Fig. S2 and S3 of the ESI† for ^1^H and ^13^C NMR spectra, respectively). Anal. Calcd for C_32_H_22_S_4_: C, 71.87; H, 4.15. Found: C, 71.58; H, 4.24.

### High-resolution X-ray crystallographic measurement of 1 (S, S)

Single crystal high-resolution data (sin(*θ*/*λ*_max_) = 1.08 Å^−1^) were collected at 100(2) K on a Rigaku FRE+ equipped with VHF Varimax confocal mirrors, an AFC10 goniometer and an HG Saturn724+ detector using Mo-Kα radiation (*λ* = 0.71075 Å). The Crystal Clear 3.1 software^34^ was used for data collection and CrysAlisPro^35^ for data reduction and Gaussian absorption correction. SORTAV^36^ was used to average and merge the sets of intensities. The crystallographic CIF file (CCDC-1811040) is provided in the ESI.†

The crystal structure was solved using direct methods and least-squares independent atom refinement (IAM) was carried out with the SHELX-2014 (ref. 37) software package. All non-hydrogen atoms were refined with anisotropic displacement parameters, while all hydrogen atoms were calculated at theoretical positions with *U*_iso_ = 1.2 (see Fig. 2 for the crystal structure of 1 (S, S) and the crystal data and refinement details in Table S1 of the ESI†). This model served as the initial point for the aspherical atom refinement, which was implemented using the Hansen–Coppens formalism^38^ as implemented in the XD2016 program.^39^ According to this formalism, electron density in a crystal is divided into three components as expressed in eqn (1):1
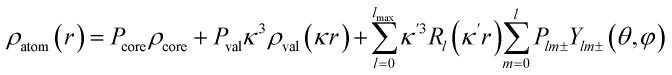
where, the first term is the spherically averaged free-atom Hartree–Fock core contribution, *ρ*_core_, with population parameter *P*_c_. The second term is the spherically averaged free atom Hartree–Fock normalized to one electron valence contribution, *ρ*_valence_, with population parameter *P*_v_, modified by the expansion/contraction parameter *κ*. The third term represents the deviation of the valence density from spherical symmetry modified by the expansion/contraction parameter *κ*′. The deformation is expressed by a density normalized Slater-type radial function 
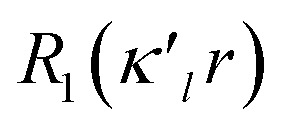
 modulated by the density normalized, real spherical harmonics angular functions *d*_*lm*±_(**r**/*r*) defined on local axes centered on the atoms and with population parameters *P*_*lm*±_.2
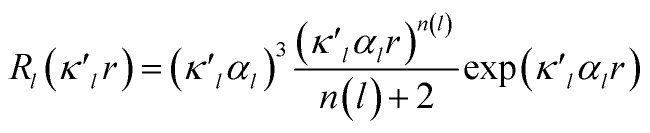



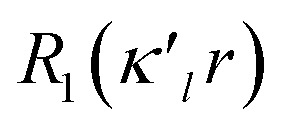
 is given by eqn (2), where *n*(*l*) ≥ 1 obeys Poisson's electrostatic equation and values for *α*_*l*_ are estimated from the Hartree–Fock optimized single-ξ exponents of the valence orbital wave function calculated for free atoms. Scattering factors for C, H and S were derived from the wave functions tabulated by Clementi and Roetti.^40^ As shown in the literature, the use of default values of *n*(*l*) = (4,4,4,4) and *α*_*l*_ for second-row atoms (P, S) may lead to ambiguous results.^38,41^ Thus, several models described previously^42,43^ were tested and finally *n*(*l*) = (4,4,6,8) values were used. An identical set of *n*(*l*) was used to refine the experimental data of another hypervalent sulfur-nitrogen species^44,45^ as well as in experimental studies of l-cysteine.^46^ The *α*_*l*_ parameter was kept constant at 3.851*a*_o_^−1^.^47^ Initially, only the scale factor was refined for all data. Next, accurate positional and displacement parameters for all non-hydrogen atoms were obtained from the high order refinement (sin(*θ*/*λ*) > 0.7 Å^−1^), while positional and isotropic displacements for hydrogen atoms were refined using low-angle data (sin(*θ*/*λ*) < 0.7 Å^−1^). Due to the unavailability of neutron data, all C–H distances were fixed to the averaged distances from neutron studies^48^ (*e.g.*, *d*_Carom–H_ = 1.083 Å). During the next stages of refinement, monopole, dipole, quadrupole, octupole and hexadecapole populations were refined with the single expansion/contraction *κ* parameter in a stepwise manner. The expansions over the spherical harmonics were truncated at the hexadecapolar level [*l*_max_ = 4] for the sulfur-bonded atoms (^A^S, ^B^S, C_*i*_, ⋯) and at the octupolar level [*l*_max_ = 3] for the remaining carbon. Hydrogen atoms were represented by the bond directed dipole. Finally, a single *κ*′ parameter was refined for all non-hydrogen atoms. Chemically and symmetry related atoms were constrained to share the same expansion/contraction (*κ*/*κ*′) parameters. Throughout, the multipole refinement expansion/contraction parameters (*κ*/*κ*′) of all hydrogen atoms were fixed to values *κ* = 1.20 and *κ*′ = 1.20. This procedure was repeated several times in a block refinement until satisfactory convergence was achieved. Chemical constraints for similar atoms were applied at the initial stages of the refinements. These constraints were gradually released and the final model was chemically unconstrained. The electron neutrality condition was imposed on the molecule for the entire refinement. Final multipole refinement led to a featureless residual density map (Fig. S4 in the ESI†). The overall residual density after multipole refinement with all high order data was −0.24 ≤ ∇^2^ and *ρ* ≤ 0.25 e Å^−3^.

The multipolar refinement details are shown in Table S1 in the ESI.† For further details, see Table S2 in the ESI.†

### Methodological details used for calculations

Calculations were performed using the Gaussian 09 program package.^49^ Compound 1 (S, S) was optimized with the 6-311+G(d) basis set for S and the 6-31G(d,p) basis sets for C and H. Herein, we refer to this basis set system as A (BSS-A). The Møller–Plesset second order energy correlation (MP2) level was applied to the calculations.^50^ The structural parameters optimized with MP2/BSS-A (*r*(^A^S, ^A^S) = 2.0730 Å and *r*(^A^S, ^B^S) = 2.9874 Å) were very close to the observed values (*r*_obsd_(^A^S, ^A^S) = 2.0559(5) Å and *r*_obsd:av_(^A^S, ^B^S) = 2.9852 Å), respectively. Compounds 1–4 were similarly optimized with MP2/BSS-A, where the 6-311+G(d) basis sets were applied to S and/or Se with the 6-31G(d,p) basis sets for C and H. The deformation density map for 1 (S, S) was computed using the Multiwfn program.^51^

The QTAIM functions were calculated using the Gaussian 09 program package^49^ with MP2/BSS-A. The results were analyzed with the AIM2000 program.^52^ Normal coordinates of internal vibrations (NIV) obtained by frequency analysis were employed to generate the perturbed structures.^20,21^ A *k*-th perturbed structure (**S**_*kw*_) was generated by the addition of the normal coordinates of the *k*-th internal vibration (**N**_*k*_) to the standard orientation of a fully optimized structure (**S**_o_) in the matrix representation as shown in eqn (3). The coefficient *f*_*kw*_ in eqn (3) was determined to satisfy eqn (4) for an interaction in question. The perturbed structures generated with NIV correspond to the structures in the zero-point internal vibrations, where the interaction distances in question are elongated or shortened to the values given in eqn (4), where *r* and *r*_o_ stand for the distances in the perturbed and fully optimized structures, respectively, with *a*_o_ the Bohr radius (0.52918 Å).^16,17,52^**N**_*k*_ significant to five decimal places was used to predict **S**_*kw*_.

3 **S**_*kw*_ = **S**_o_ + *f*_*kw*_**N**_*k*_

4 *r* = *r*_o_ + *wa*_o_, (*w* = (0), ±0.05 and ±0.1; *a*_o_ = 0.52918 Å)

5 *y* = *c*_o_ + *c*_1_*x* + *c*_2_*x*^2^ + *c*_3_*x*^3^, (*R*_c_^2^: square of correlation coefficient)

*H*_b_(***r***_c_) *versus H*_b_(***r***_c_) − *V*_b_(***r***_c_)/2 was plotted for data from five points of *w* = 0, ±0.05 and ±0.1 in eqn (4) in QTAIM-DFA. Each plot for an interaction was analyzed using a regression curve assuming the cubic function in eqn (5), where (*x*, *y*) = (*H*_b_(***r***_c_) − *V*_b_(***r***_c_)/2, *H*_b_(***r***_c_)) (*R*_c_^2^ > 0.99999).^18–22,53^

## Results and discussion

### High-resolution X-ray diffraction determination of electron densities for 1 (S, S)

**Fig. 3:**
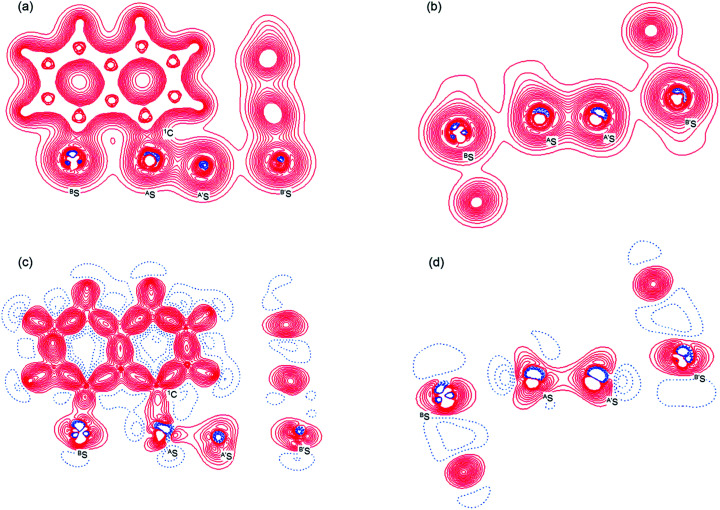
Valence electron density map drawn on the ^B^S^A^SC_1_ plane of 1 (S, S) and the magnified map for the ^B^S⋯^A^S–^A′^S⋯^B′^S interaction drawn on the ^B^S^A^S^A′^S plane ((a) and (b), respectively), in which the contour level is 0.1 e Å^−3^. Deformation density map drawn on the ^B^S^A^SC_1_ plane of 1 (S, S) and the magnified map for the ^B^S⋯^A^S–^A′^S⋯^B′^S interaction drawn on the ^B^S^A^S^A′^S plane ((c) and(d), respectively), in which contour level is 0.05 e Å^−3^. The red and blue lines correspond to the increased and decreased electron densities, respectively, in the formation of the chemical bonds or interactions.

**Fig. 4:**
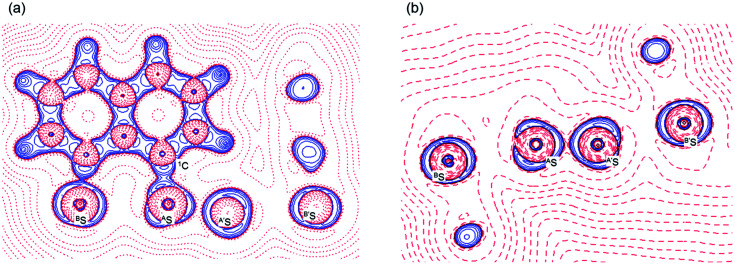
Positive Laplacian map in the ^B^S^A^SC_1_ plane of 1 (S, S) (a) and magnified ^B^S⋯^A^S–^A′^S⋯^B′^S interaction region in the ^B^S^A^S^A′^S plane (b). Positive and negative areas are shown by red and blue lines, respectively and each contour level is 0.05 e Å^−3^.

**Fig. 5:**
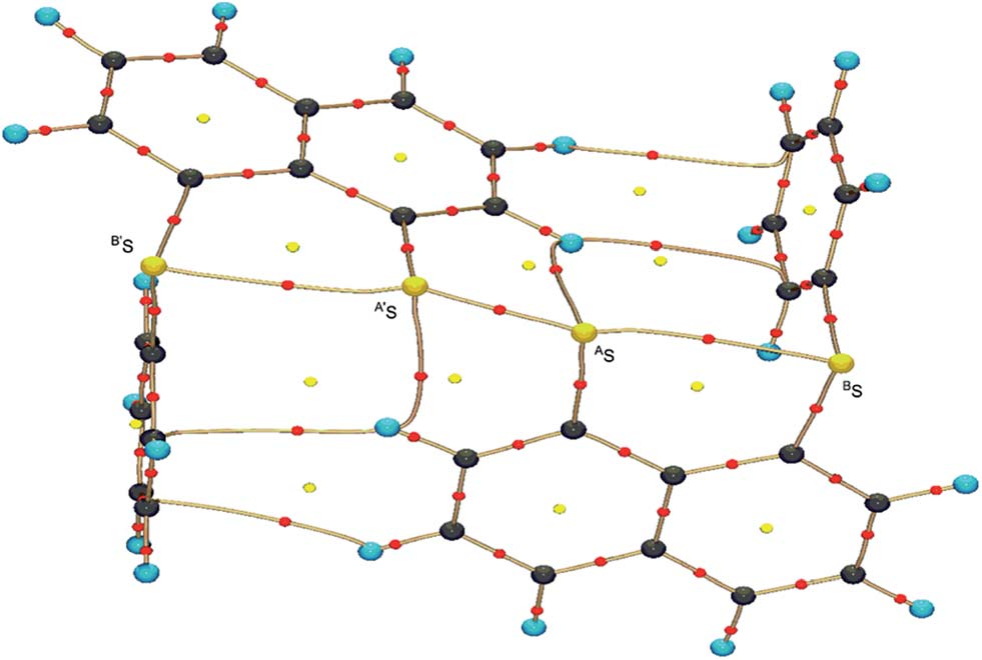
Molecular graph of 1 (S, S) determined by high-resolution X-ray crystallographic analysis.

Fig. 2 shows the crystal structure of 1 (S, S) determined by high-resolution X-ray crystallographic analysis and Table 1 shows selected bond distances, angles and torsional angles. The *r*_obsd_(^A^S, ^A′^S) and *r*_obsd:av_(^A^S, ^B^S) values were determined to be 2.0559(8) Å (ref. 54) and 2.9852(8) Å, respectively, with ∠^B^S^A^S^A′^S_obsd:av_ of 167.2° for 1 (S, S). The ^B^S⋯^A^S–^A′^S⋯^B’^S interaction in 1 (S, S) can be described as linear since ∠^B^S^A^S^A′^S is larger than 150°, which is the cut-off value we propose to determine the linearity of these interactions. Accordingly, the ^B^S⋯^A^S–^A′^S⋯^B′^S interaction can be well described by the S_4_ σ(4c–6e) model. Fig. 3 depicts the valence electron density map in the ^A′^S^A^SC_1_ plane for 1 (S, S) with magnification around the ^B^S⋯^A^S–^A′^S⋯^B′^S interaction in the ^B^S^A^S^A′^S (^B′^S) plane (Fig. 3b). The deformation density map in the ^A′^S^A^SC_1_ plane for 1 (S, S) and the magnified map around the ^B^S⋯^A^S–^A′^S⋯^B′^S interaction in the ^B^S^A^S^A′^S (^B′^S) plane are presented in Fig. 3c and d, respectively. The positive Laplacian map in the ^A′^S^A^SC_1_ plane for 1 (S, S) and the magnified map around the ^B^E⋯^A^E–^A^E⋯^B^E interaction in the ^B^S^A^S^A′^S (^B′^S) plane are presented in Fig. 4a and b, respectively. The BCPs around ^B^S-∗-^A^S-∗-^A′^S-∗-^B′^S in 1 (S, S) are expected to be located in the negative area of ∇^2^*ρ*_b_(***r***_c_). Fig. 5 illustrates the molecular graph of 1 (S, S), which was determined by high-resolution X-ray crystallographic analysis. All the BCPs are detected as expected, including those around ^B^S-∗-^A^S-∗-^A′^S-∗-^B′^S in 1 (S, S). Two pairs of BPs with BCPs are also detected for the weaker interactions, which are very close to those drawn theoretically and therefore discussed in the theoretical section.

**Table 1:** Selected structural parameters observed for 1 (S, S) and those evaluated with MP2/BSS-A^a^

Species (^A^S, ^B^S) (symmetry)	*r*(^A^S, ^A′^S) (Å)	Δ*r*(^A^S, ^A′^S)^b^ (Å)	*r*(^A^S, ^B^S) (Å)	Δ*r*(^A^S, ^B^S)^b^ (Å)	Δ*r*_van_(^A^S, ^B^S)^c^ (Å)	∠^A′^S^A^SC_1_ (°)	∠C_8_^B^SC_*i*_ (°)	∠^B^S^A^S^A′^S (°)	*ϕ*_1_ d (°)	*ϕ*_2_ e (°)
1 (S, S) (*C*_1_)_obsd_	2.0559(5)	0.000	2.9852^f^	0.000	−0.615	105.0^g^	102.5^h^	167.2^i^	−89.5	−75.6^j^
1 (S, S) (*C*_2_)_calcd_	2.0730	0.017	2.9874	0.002	−0.613	104.0	100.6	169.4	−78.2	−64.2

a BSS-A; the 6-311+G(d) basis set for S with the 6-31G(d,p) basis sets for C and H.

b Δ*r*(^A^S, ^X^S) = *r*_calcd_(^A^S, ^X^S) − *r*_obsd_(^A^S, ^X^S), where *X* = A′ and B.

c Δ*r*_van_(^A^S, ^B^S) = *r*(^A^S, ^B^S) − ∑*r*_vdW_(^A^S, ^B^S), where *r*_vdW_(S) = 1.80 Å (ref. 55).

d *ϕ*_1_ = *ϕ*(C_1_^A^S^A′^SC_1′_).

e *ϕ*_2_ = *ϕ*(C_9_C_8_^B^SC_*i*_) and/or *ϕ*(C_19_C_18_^B′^SC_*i*′_).

f Averaged value: *r*_obsd:av_(^A^S, ^B^S) = 2.9879(4) Å and 2.9825(5) Å.

g Averaged value: ∠C_1_^A^S^A′^S_obsd:av_ = 105.54(2)° and 104.48(2)°.

h Averaged value: ∠C_8_^B^SC_*i*-obsd:av_ = 101.94(1)° and 102.99(2)°.

i Averaged value: ∠^B^S^A^S^A′^S_obsd:av_ = 168.68(2)° and 165.70(2)°.

j Averaged value: *ϕ*_2-obsd:av_ = −70.22(2)° and −80.98(2)°.

### Formation of S_4_ σ(4c–6e) in 1 (S, S) confirmed based on experimental background

The electron distributions can be clearly observed in Fig. 3. The valence electron density map of 1 (S, S) seems to define (three-dimensional) saddle points of *ρ*(***r***) between ^A^S and ^B^S and between ^A′^S and ^B′^S of 1 (S, S) and the typical saddle point between ^A^S and ^A′^S (see Fig. 3a or b). Each saddle point of *ρ*(***r***) between the adjacent S atoms in ^B^S⋯^A^S–^A′^S⋯^B′^S should correspond to a BCP on a BP in 1 (S, S) (see also Fig. 5). The enhanced charge density at ^B^S directs toward to the depleted area at ^A^S extending over the backside of the ^A^S–^A′^S bond, as shown in Fig. 3c and d. This shows the contribution of the CT interaction of the n_p_(^B^S)→σ∗(^A^S–^A′^S) form. Similar phenomena can be found between ^B′^S and ^A′^S–^A^S, which show the CT interaction of the n_p_(^B′^S)→σ∗(^A′^S–^A^S) form. Such degenerated CT interactions should be analyzed as S_4_ σ(4c–6e) of the n_p_(^B^S)→σ∗(^A^S–^A′^S)←n_p_(^B′^S) form, which must be the driving force for the formation of S_4_ σ(4c–6e) as proposed by our group. The valence electron density maps and the deformation density maps around the ^B^S⋯^A^S–^A′^S⋯^B′^S interaction, as shown in Fig. 3, strongly support the formation of linear S_4_ σ(4c–6e) of the n_p_(^B^S)→σ∗(^A^S–^A′^S)←n_p_(^B′^S) type in 1 (S, S) based on the experimental treatment.

As shown in Fig. 4, three VSCCs (valence shell charge concentrations) appear at each S atom in the ^B^S^A^S^A′^S (^B′^S) plane of 1 (S, S). A pair of VSCCs on ^A^S and ^B^S is beginning to merge with each other, which confirms the presence of the ^A^S⋯^B^S interaction. The ^A′^S⋯^B′^S interaction is similarly confirmed through almost merging between the VSCCs on ^A′^S and ^B′^S. These results together with the original ^A^S–^A′^S bond also confirm the formation of S_4_ σ(4c–6e) in 1 (S, S). The linearity of the VSCCs is not very good, which would affect the BPs between the atoms. The differences between the lengths of the BPs (*r*_BP_) and the straight-line distances (*R*_SL_) (Δ*r*_BP_ = *r*_BP_ − *R*_SL_) are less than 0.0010 Å and 0.012–0.013 Å for ^A^S–^A′^S and ^A^S⋯^B^S (and ^A′^S⋯^B′^S), respectively, in 1 (S, S). Therefore, each of the ^B^S⋯^A^S–^A′^S⋯^B′^S interactions in 1 (S, S) can be approximated to a linear interaction.

The BCPs on BPs around ^B^S⋯^A^S–^A′^S⋯^B′^S in 1 (S, S) are clearly specified in the molecular graph drawn experimentally in Fig. 5 together with that expected for 1 (S, S). Some QTAIM parameters were experimentally determined at the BCPs around ^B^S⋯^A^S–^A′^S⋯^B′^S in 1 (S, S) (see the observed values for the QTAIM parameters and that evaluated theoretically with MP2/BSS-A employing the observed structure of 1 (S, S) in Table 2). Although the ^A^S–^A′^S bond in 1 (S, S) is experimentally classified by the regular CS (r-CS) interactions, the ^A^S⋯^B^S and ^A′^S⋯^B′^S interactions are shown to exist just on the border area between the pure CS (p-CS) and r-CS interactions (see, Fig. 5 and Table 2). The values evaluated theoretically employing the observed structure of 1 (S, S) reproduced the experimentally obtained values accurately except for (*ħ*^2^/8*m*)∇^2^*ρ*_b_(***r***_c_) (= *H*_b_(***r***_c_) − *V*_b_(***r***_c_)/2), *H*_b_(***r***_c_) and *k*_b_(***r***_c_) (= *V*_b_(***r***_c_)/*G*_b_(***r***_c_)) at the BCP of the ^A^S–^A′^S bond although this deviation does not seem to be very severe. However, the difference in (*ħ*^2^/8*m*)∇^2^*ρ*_b_(***r***_c_) (= *H*_b_(***r***_c_) − *V*_b_(***r***_c_)/2) will greatly affect the classification of ^A^S–^A′^S since the signs are opposite to the values predicted experimentally and the value calculated employing the observed structure of 1 (S, S).

**Table 2:** QTAIM functions and QTAIM-DFA parameters for ^A^E-∗-^A′^E and ^A^E-∗-^B^E at BCPs of 1-(8-Ph^B^EC_10_H_6_)^A^E–^A′^E(C_10_H_6_^B^EMe-8′)-1′ (1–4)^a^

Species (symmetry)	Interaction (X-∗-Y)	*ρ*_b_(***r***_c_) (*ea*_o_^−3^)	*c*∇^2^*ρ*_b_(***r***_c_)^b^ (au)	*H*_b_(***r***_c_) (au)	*k*_b_(***r***_c_)^c^	*R* (au)	*θ* (°)	*ν*_*n*_ (*n*)^d^ (cm^−1^)	*k*_f_ e (mDyn Å^−1^)	*θ*_p_ (°)	*κ*_p_ (au^−1^)	Classification/characterization
1 (*C*_1_)_obsd_^f^	(^A^S-∗-^A′^S)	0.141	0.004	−0.11	−1.92							r-CS
	(^A^S-∗-^B^S)	0.020	0.008	0.00	−1.00							p-CS/r-CS
	(^A′^S-∗-^B′^S)	0.021	0.008	0.00	−1.00							p-CS/r-CS
1 (*C*_1_)_obsd_^g^	(^A^S-∗-^A^S)	0.1418	−0.0111	−0.0748	−2.424	0.0757	188.5					SS
	(^A^S-∗-^B^S)	0.0229	0.0075	−0.0004	−1.027	0.0075	93.1					r-CS
	(^A′^S-∗-^B′^S)	0.0234	0.0076	−0.0005	−1.031	0.0076	93.7					r-CS
1 (*C*_2_)_calcd_	(^A^S-∗-^A^S)	0.1373	−0.0097	−0.0697	−2.383	0.0704	187.9	518.7 (48)	1.701	197.5	0.5	SS/Cov-w
	(^A^S-∗-^B^S)	0.0227	0.0075	−0.0004	−1.026	0.0075	93.1	181.1 (17)	0.209	117.8	68.9	r-CS/*t*-HB-wc^h^
2 (*C*_2_)_calcd_	(^A^S-∗-^A^S)	0.1356	−0.0089	−0.0677	−2.354	0.0683	187.5	502.9 (48)	1.698	197.5	0.6	SS/Cov-w
	(^A^S-∗-^B^Se)	0.0225	0.0068	−0.0006	−1.042	0.0069	95.1	152.0 (16)	0.154	128.1	133.3	r-CS/*t*-HB-wc^h^
3 (*C*_2_)_calcd_	(^A^Se-∗-^A^Se)	0.0970	−0.0018	−0.0403	−2.095	0.0404	182.5	288.9 (28)	0.442	186.6	2.5	SS/Cov-w
	(^A^Se-∗-^B^S)	0.0246	0.0070	−0.0011	−1.071	0.0070	93.7	150.9 (15)	0.086	140.1	126.4	r-CS/*t*-HB-wc^h^
4 (*C*_2_)_calcd_	(^A^Se-∗-^A^Se)	0.0948	−0.0013	−0.0387	−2.070	0.0387	181.9	275.5 (28)	0.664	187.1	2.4	SS/Cov-w
	(^A^Se-∗-^B^Se)	0.0250	0.0064	−0.0014	−1.098	0.0066	102.3	126.0 (15)	0.105	150.5	141.8	r-CS/CT-MC

a The 6-311+G(d) basis set was employed for S and Se with the 6-31G(d,p) basis set for C and H.

b *c*∇^2^*ρ*_b_(***r***_c_) = *H*_b_(***r***_c_) − *V*_b_(***r***_c_)/2, where *c* = *ℏ*^2^/8*m*.

c *k*_b_(***r***_c_) = *V*_b_(***r***_c_)/*G*_b_(***r***_c_).

d Corresponding to the interaction in question. Symmetric and anti-symmetric modes being employed for ^A^E-∗-^A′^E and ^A^E-∗-^B^E, respectively.

e Force constant for *ν*_*n*_.

f Observed values for QTAIM parameters.

g Calculated values for QTAIM parameters evaluated employing the observed structure.

h Typical HB nature with covalency.

### Theoretical basis for the nature of S_4_ σ(4c–6e) in 1 (S, S)

#### Structure of 1 (S, S) optimized at the MP2 level

Compound 1 (S, S) was optimized with MP2/BSS-A, retaining *C*_2_ symmetry. Table 1 shows selected predicted bond distances, angles and torsional angles for 1 (S, S). The predicted structural parameters remarkably match the corresponding observed parameters. The *r*_calcd_(^A^E, ^A^E) and *r*_calcd_(^A^E, ^B^E) values are 2.0730 and 2.9874 Å, respectively. The differences between the calculated and observed distances for ^A^E–^A^E and ^A^E⋯^B^E are also given in Table 1, which are defined as Δ*r*(^A^E, ^A^E) = *r*_calcd_(^A^E, ^A^E) − *r*_obsd_(^A^E, ^A′^E) and Δ*r*(^A^E, ^B^E) = *r*_calcd_(^A^E, ^B^E) − *r*_obsd_(^A^E, ^B^E), respectively. The Δ*r*(^A^E, ^A^E) and Δ*r*(^A^E, ^B^E) values are 0.017 and 0.002 Å, respectively. The Δ*r*(^A^E, ^B^E) value of 0.002 Å completely satisfies this criterion, while the Δ*r*(^A^E, ^A^E) value of 0.017 Å is only slightly larger than 0.013 Å. The magnitude of Δ*r* less than 0.013 Å is desirable for QTAIM-DFA, which corresponds to a half of each interval of adjacent two data points in the plots of *H*_b_(***r***_c_) *versus H*_b_(***r***_c_) − *V*_b_(***r***_c_)/2 in QTAIM-DFA (0.013 Å = 0.05*a*_o_/2 = 0.026 Å/2; see Fig. 8). Despite the slightly larger magnitude of Δ*r*(^A^E, ^A′^E), the optimized overall structure of 1 (S, S) can be considered to satisfy the criterion since the ^A^E⋯^B^E interactions are the principle concern for S_4_ σ(4c–6e).

#### Deformation density map around ^B^S-∗-^A^S-∗-^A^S-∗-^B^S of 1 (S, S)

**Fig. 6:**
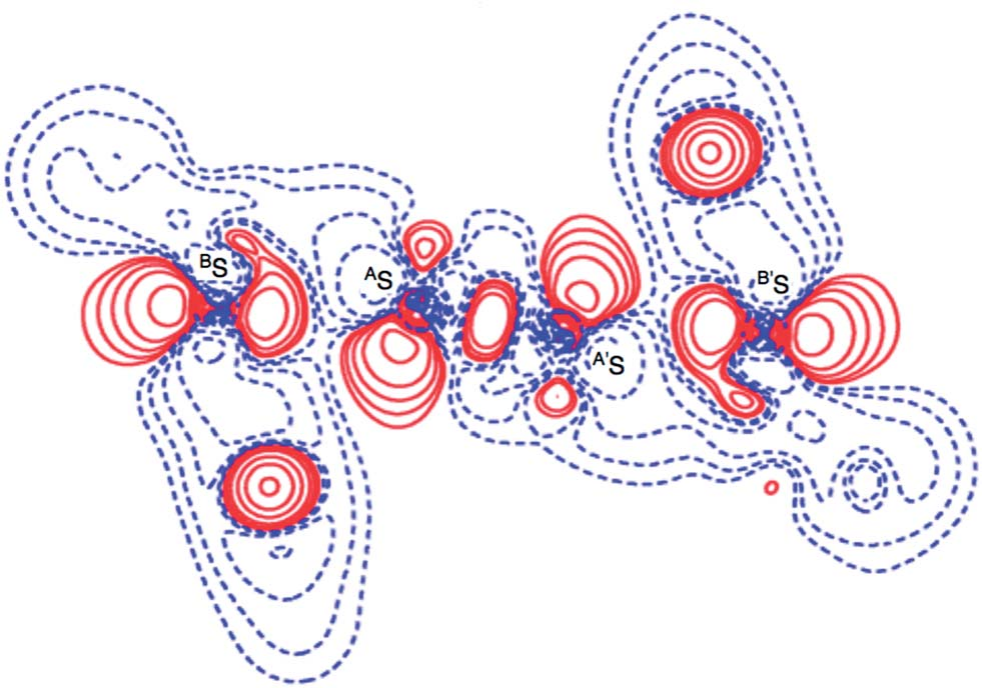
Deformation density map for the ^B^S⋯^A^S–^A′^S⋯^B′^S interaction drawn on the ^B^S^A^S^A′^S plane of 1 (S, S), of which contour level is 0.05 e Å^−3^. The red and blue lines correspond to the increased and decreased electron densities, respectively, in the formation of the chemical bonds or interactions.

The deformation density map was drawn theoretically on the ^B^S^A^S^A′^S plane around the ^B^S-∗-^A^S-∗-^A′^S-∗-^B′^S interaction of 1 (S, S) in *C*_2_ symmetry, similarly to the case of the experimental approach as shown in Fig. 6. The deformation density map shown in Fig. 6 is (very) similar to that shown in Fig. 3d. The enhanced charge density at ^B^S also directs toward to the depleted area at ^A^S extending over the backside of the ^A^S–^A′^S bond, as shown in Fig. 6. In particular, the CT interaction of the n_p_(^B^S)→σ∗(^A^S–^A′^S)←n_p_(^B′^S) type is also demonstrated theoretically by the deformation density map, which should be analyzed as linear S_4_ σ(4c–6e) as discussed above. The contribution of the CT interaction of the n_p_(^B^E)→σ∗(^A^E–^A^E)←n_p_(^B^E) type in 1 (^A^E = ^B^E = S) was evaluated by the second order perturbation of the Fock matrix (*E*(2))^56^ with MP2/BSS-A, and also those for 2–4 (^A^E, ^B^E = S and/or Se). The results are shown in Table S4 of the ESI.† The *E*(2) values of 13.2–26.1 kcal mol^−1^ were predicted for 1–4 depending on ^A^E and ^B^E, where σ∗(^A^E–^A^E) contributes much more than the case of n(^B^E). The S_4_ σ(4c–6e) nature of the linear S_4_ interaction in 1 (S, S) as well as in ^A^E_2_^B^E_2_ σ(4c–6e) in 2–4 (^A^E, ^B^E = S and/or Se) are theoretically well established.

#### Molecular graph, contour plot, and negative Laplacian around ^B^S-∗-^A^S-∗-^A′^S-∗-^B′^S in 1 (S, S)

**Fig. 7:**
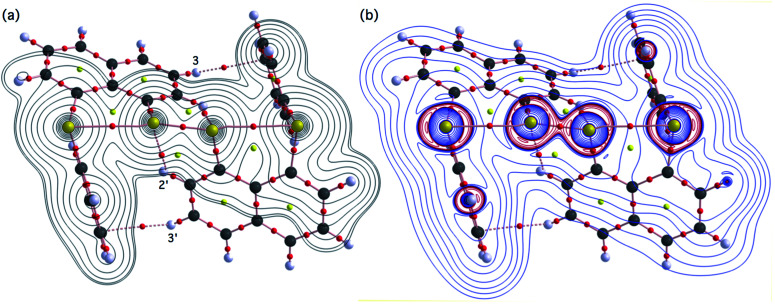
Molecular graphs for 1 (S, S) with contour plot (a) and negative Laplacian map (b) calculated with MP2/BSS-A. BCPs (bond critical points) are denoted by red dots, RCPs (ring critical points) by yellow dots and CCPs (cage critical points) by green dots. BPs (bond paths) are drawn as pink lines and the secondary one as pink dots. They are associated with the BCPs. Carbon, hydrogen and sulfur atoms are shown in black, gray and yellow, respectively. The contours (*ea*_0_^−3^) for (a) are at 2^*l*^ (*l* = ±8, ±7 … and 0) with 0.0047 (bold line). Positive and negative areas in (b) are in blue and red lines, respectively.

Fig. 7 shows the molecular graph, contour plot, and negative Laplacian map for 1 (S, S) calculated with MP2/BSS-A. All BCPs are detected as expected, including those around ^B^S-∗-^A^S-∗-^A′^S-∗-^B′^S in 1 (S, S). Similarly to the case of the experimental results, BCPs are also detected for the weaker interactions of ^A^S-∗-^2′^H with ^A′^S-∗-^2^H and ^3^H-∗-^*p*′^C with ^3′^H-∗-^*p*^C. The former may play an additional role in stabilising the linear ^B^S-∗-^A^S-∗-^A′^S-∗-^B′^S interaction, while the latter is likely to influence the specific positions of the phenyl groups through the C_Nap_–H⋯π(C_6_H_5_S) interactions. As shown in Fig. 7b, the BCP on ^A^S-∗-^A′^S is located in the negative area of ∇^2^*ρ*_b_(***r***_c_), while those on ^A^S-∗-^B^S are in the positive region. These results indicate that ^A^S-∗-^A′^S and ^A^S-∗-^B^S are classified by shared shell (SS) and closed shell (CS) interactions, respectively. Accordingly, these results clearly demonstrate the formation of S_4_ σ(4c–6e) in 1 (S, S) theoretically. As shown in Fig. 7, the BPs (^A^S-∗-^B^S) seem somewhat bent around ^A^S. However, the differences between *r*_BP_ and the corresponding *R*_SL_ (Δ*r*_BP_ = *r*_BP_ − *R*_SL_) are 0.001 Å and 0.021 Å for Δ*r*_BP_ (^A^S, ^A′^S) and Δ*r*_BP_ (^A^S, ^B^S), respectively, in 1 (S, S) (Table S3 of the ESI†). These results indicate that each of the ^B^S⋯^A^S–^A′^S⋯^B′^S interactions in 1 (S, S) can also be approximated as a linear interaction theoretically. The Δ*r*_BP_ values in 5–8 have been discussed elsewhere.^11^

The nature of the non-covalent E⋯E interaction was established for E = S in 1 (S, S) both experimentally and theoretically in this study. The interaction becomes much stronger for E = Se in 4 (Se, Se) based on the theoretical investigations. The results are in accordance with those reported recently^14–16^ although the strength of S⋯S seems to change widely as the structure changes.

#### Application of QTAIM-DFA to ^A^E_2_^B^E_2_ σ(4c–6e) (^A^E, ^B^E = S, Se)

**Fig. 8:**
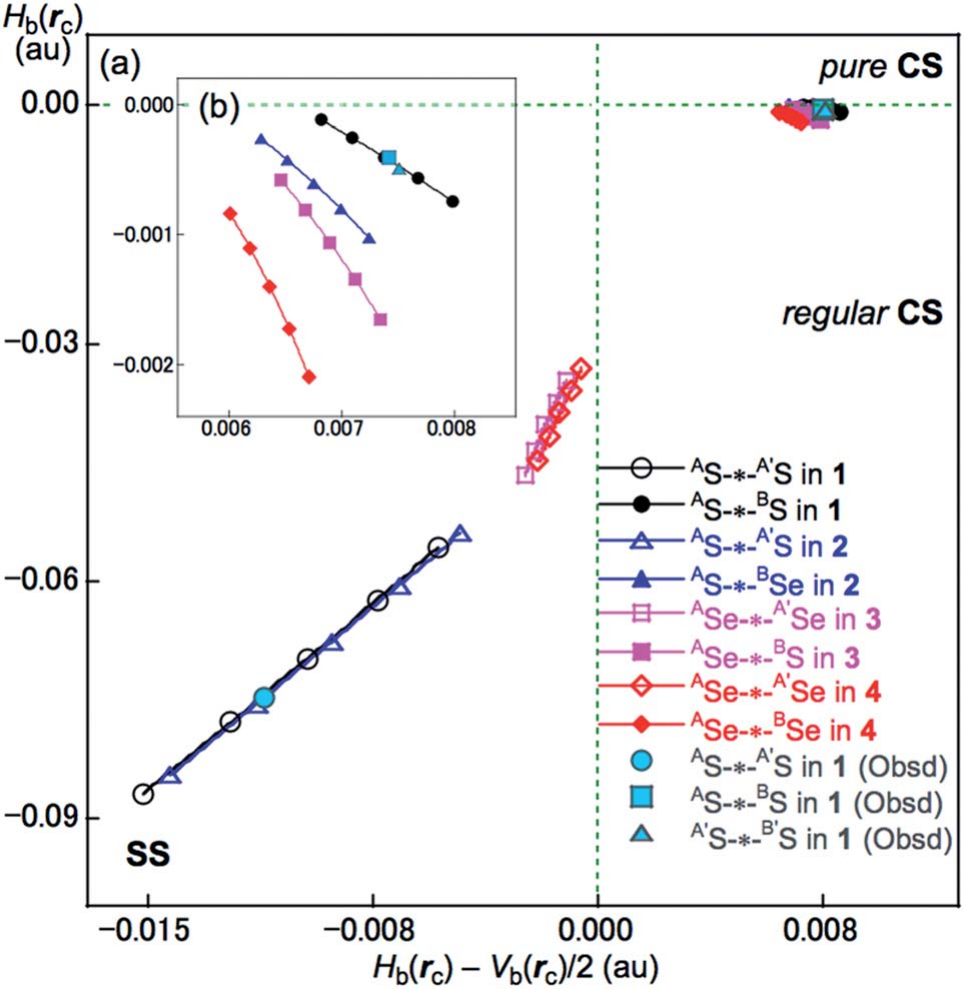
Plots of *H*_b_(***r***_c_) *versus H*_b_(***r***_c_) − *V*_b_(***r***_c_)/2 for ^A^E-*-^A^E and ^A^E-*-^B^E of 1–4. (a) Whole plot and (b) magnified plot for ^A^E-*-^B^E. Marks and colours for the species are shown in the figure.

The QTAIM functions of *ρ*_b_(***r***_c_), *H*_b_(***r***_c_) − *V*_b_(***r***_c_)/2, *H*_b_(***r***_c_) and *k*_b_(***r***_c_) (= *V*_b_(***r***_c_)/*G*_b_(***r***_c_)) were evaluated for ^A^E-∗-^A^E and ^A^E-∗-^B^E at the BCPs and Table 2 presents the values obtained. Table 2 also contains the frequencies (*ν*) and force constants (*k*_f_) corresponding to ^A^E-∗-^A′^E and ^A^E-∗-^B^E. Fig. 8 shows the plots of *H*_b_(***r***_c_) *versus H*_b_(***r***_c_) − *V*_b_(***r***_c_)/2 for the fully optimized data of 1–4 given in Table 2 together with those from the perturbed structures around the fully optimized structures; the perturbed structures were generated with NIV according to eqn (3) and eqn (4). The plots were analyzed according to eqn (S3)–(S6) in the ESI† and the QTAIM-DFA parameters of (*R*, *θ*) and (*θ*_p_, *κ*_p_) were obtained. Table 2 presents the (*R*, *θ*) and (*θ*_p_, *κ*_p_) values for ^A^E-∗-^A^E and ^A^E-∗-^B^E in 1–4.

#### Nature of ^A^S-∗-^A^S and ^A^S-∗-^B^S in 1 (S, S) as defined by *θ* and *θ*_p_

The nature of ^A^S-∗-^A^S and ^A^S-∗-^B^S in 1 (S, S) was determined using the QTAIM-DFA parameters of (*R*, *θ*) and (*θ*_p_, *κ*_p_) with the standard values in Scheme S2 of the ESI† as a reference. The *θ* values are for the classification of interactions, which can then be characterized by *θ*_p_ with *R* being used to sub-divide the covalent interactions. It is instructive to survey the criteria briefly in relation to this study. The CS and SS interactions have values of 45° < *θ* < 180° (0 < *H*_b_(***r***_c_) − *V*_b_(***r***_c_)/2) and 180° < *θ* < 206.6° (*H*_b_(***r***_c_) − *V*_b_(***r***_c_)/2 < 0), respectively. The CS interactions are sub-divided into p-CS and r-CS for values of 45° < *θ* < 90° (0 < *H*_b_(***r***_c_)) and 90° < *θ* < 180° (*H*_b_(***r***_c_) < 0), respectively. The *θ*_p_ value plays an important role in characterizing the interactions. In the p*-*CS region of 45° < *θ* < 90°, when 45° < *θ*_p_ < 90°, the interactions will be vdW type character, while for 90° < *θ*_p_ < 125°, they will be typical HB (*t*-HB)-type without covalency (*θ*_p_ of 125° corresponds to *θ* = 90°).^18–21^ CT interactions will occur in the r-CS region, where 90° < *θ* < 180°. The *t*-HB interactions with covalency (*t*-HB-wc) appear in the range of 125° < *θ*_p_ < 150° (90° < *θ* < 115°). CT-MC (molecular complex through charge transfer) and CT-TBP (trigonal bipyramidal adduct through CT) type interactions will appear in the ranges of 150° < *θ*_p_ < 180° (115° < *θ* < 150°) and 180° < *θ*_p_ < 190° (150° < *θ* < 180°), respectively, although CT-TBP was not observed in this study. *R* provides a way to sub-classify the SS interactions, where the classical covalent SS chemical bonds are weak (Cov-w) when *R* < 0.15 au.

The (*θ*, *θ*_p_, *R*) values for ^A^S-∗-^A^S in 1 (S, S) are (187.9°, 197.5°, 0.0704 au), which is therefore classified as SS and predicted to have weak covalent nature (SS/Cov-w). Similarly, (*θ*, *θ*_p_, *R*) = (93.1°, 117.8°, 0.0075 au) for ^A^S-∗-^B^S, which is therefore classified as r-CS. The interaction is predicted to have *t*-HB-wc nature irrespective of *θ*_p_ = 117.8° (<125°) because the *θ* value of 93.1° (>90°) is superior to *θ*_p_ = 117.8° (<125°) [*θ*_p_ = 125° corresponds to *θ* = 90° for typical interactions (see Scheme S2 of the ESI†)]. Therefore, ^A^S-∗-^B^S in 1 (S, S) is predicted to have r-CS/*t*-HB-wc nature overall. The nature of ^A^E-∗-^A^E and ^A^E-∗-^B^E was also predicted for 2–4. The ^A^E-∗-^A^E interactions in 2–4 have SS/Cov-w nature irrespective of the *θ*_p_ value of about 187° for 3 (Se, S) and 4 (Se, Se) since *θ* values (larger than 180°) are superior to the *θ*_p_ values in the classification. Although ^A^E-∗-^B^E in 2–4 are classified as r-CS interactions based on the criterion of 93° < *θ* < 102°, they are predicted to be of *t*-HB-wc, *t*-HB-wc and CT-MC nature, respectively, based on the *θ*_p_ values. These results are summarized in Table 2.

## Conclusion

The high-resolution X-ray diffraction determination of electron densities supported by a rigorous theoretical treatment was performed for 1 (S, S). The valence electron density map exhibits (three-dimensional) saddle points of *ρ*(***r***) between ^A^S and ^B^S and between ^A′^S and ^B′^S. Enhanced charge densities at ^B^S and ^B′^S direct toward to the depleted area around ^A^S and ^A′^S, respectively, and extend over the backside of the ^A^S–^A′^S bond. The results demonstrate the formation of S_4_ σ(4c–6e) of the n_p_(^B^S)→σ∗(^A^S–^A′^S)←n_p_(^B′^S) type. This is supported by the valence electron density map(s) and the deformation density maps in the region around ^B^S⋯^A^S–^A′^S⋯^B′^S. A pair of VSCCs originating from ^A^S and ^B^S merge with each other confirming the presence of the ^A^S⋯^B^S interaction as well as those on ^A′^S and ^B′^S forming the ^A′^S⋯^B′^S interaction. These results together with the conventional ^A^S–^A′^S bond confirm the formation of S_4_ σ(4c–6e) in 1 (S, S). The formation of S_4_ σ(4c–6e) is experimentally demonstrated clearly by BPs with BCPs in the molecular graph for ^B^S⋯^A^S–^A′^S⋯^B′^S. The ^A^S⋯^B^S and ^A′^S⋯^B′^S interactions are observed on the border area between the p-CS and r-CS interactions. These experimental results are well supported and rationalised by complementary theoretical calculations.

The dual experimental–theoretical approach provides a solid basis for understanding the behavior of ^B^E⋯^A^E–^A′^E⋯^B′^E of E_4_ σ(4c–6e) not only in 1 (S, S) but also in 2–4. This methodology has previously been applied to 2-(2-pyridylimino)-2*H*-1,2,4-thiadiazolo[2,3-*a*]pyridine,^57^ for which the behavior of N–E–N σ(3c–4e) (E = S, Se and Te) was clarified. Compilation of these results makes it possible to confirm the real existence and chemistry of hypervalent and extended hypervalent interactions.

## Conflicts of interest

The authors declare no conflict of interest.

## Supplementary Material

RA-008-C7RA13636F-s001

RA-008-C7RA13636F-s002
