# Supplementary material for: High-resolution X-ray diffraction determination of the electron density of 1-(8-PhSC10H6)SS(C10H6SPh-8′)-1′ with the QTAIM approach: evidence for S4 σ(4c–6e) at the naphthalene peri-positions
Source: RSC Adv. 2018 Mar 5;8(18):9651–60. doi: 10.1039/c7ra13636f (PMC9078645; doi:10.1039/c7ra13636f)
Supplement: RA-008-C7RA13636F-s001 [file RA-008-C7RA13636F-s001.pdf]

Electronic Supplementary Material (ESI) for RSC Advances.
This journal is © The Royal Society of Chemistry 2018

Electronic Supplementary Information

High-resolution X-ray diffraction determination of electron densities of 1-(8-$PhSC_{10}H_6$)SS($C_{10}H_6$SPh-8')-1' with QTAIM approach: Evidence for S_4 σ(4c–6e) at naphtalene *peri*-positions

Yutaka Tsubomoto,[a] Satoko Hayashi,*[,a] Waro Nakanishi,*[,a] Lucy A. Mapp[b] and Simon J. Coles*[,b]

[a] *Department of Material Science and Chemistry, Faculty of Systems Engineering, Wakayama University, 930 Sakaedani, Wakayama 640-8510, Japan. Fax: +81 73 457 8253; Tel: +81 73 457 8252; E-mail: nakanisi@sys.wakayama-u.ac.jp and hayashi3@sys.wakayama-u.ac.jp.*

[b] *Chemistry, Faculty of Natural and Environmental Sciences, University of Southampton, Southampton, SO17 1BJ. UK. Tel: +44 (0)2380596721; E-mail: S.J.Coles@soton.ac.uk.*

QTAIM Dual Functional Analysis (QTAIM-DFA)

The bond critical point (BCP; ∗) is an important concept in QTAIM. The BCP of $(\omega, \sigma) = (3, -1)$[S1] is a point along the bond path (BP) at the interatomic surface, where charge density $\rho(\boldsymbol{r})$ reaches a minimum. It is donated by $\rho_b(\boldsymbol{r}_c)$. While the chemical bonds or interactions between A and B are denoted by A–B, which correspond to BPs between A and B in QTAIM, A-∗-B emphasizes the presence of BCP (∗) in A–B.

The sign of the Laplacian $\rho_b(\boldsymbol{r}_c)$ ($\nabla^2\rho_b(\boldsymbol{r}_c)$) indicates that $\rho_b(\boldsymbol{r}_c)$ is depleted or concentrated with respect to its surrounding, since $\nabla^2\rho_b(\boldsymbol{r}_c)$ is the second derivative of $\rho_b(\boldsymbol{r}_c)$. $\rho_b(\boldsymbol{r}_c)$ is locally depleted relative to the average distribution around $\boldsymbol{r}_c$ if $\nabla^2\rho_b(\boldsymbol{r}_c) > 0$, but it is concentrated when $\nabla^2\rho_b(\boldsymbol{r}_c) < 0$. Total electron energy densities at BCPs ($H_b(\boldsymbol{r}_c)$) must be a more appropriate measure for weak interactions on the energy basis.[S1–S6] $H_b(\boldsymbol{r}_c)$ are the sum of kinetic energy densities ($G_b(\boldsymbol{r}_c)$) and potential energy densities ($V_b(\boldsymbol{r}_c)$) at BCPs, as shown in eqn (S1). Electrons at BCPs are stabilized when $H_b(\boldsymbol{r}_c) < 0$, therefore, interactions exhibit the covalent nature in this region, whereas they exhibit no covalency if $H_b(\boldsymbol{r}_c) > 0$, due to the destabilization of electrons at BCPs under the conditions.[S1] Eqn (S2) represents the relation between $\nabla^2\rho_b(\boldsymbol{r}_c)$ and $H_b(\boldsymbol{r}_c)$, together with $G_b(\boldsymbol{r}_c)$ and $V_b(\boldsymbol{r}_c)$, which is closely related to the virial theorem.

$$H_b(\boldsymbol{r}_c) = G_b(\boldsymbol{r}_c) + V_b(\boldsymbol{r}_c) \quad \text{(S1)}$$

$$(\hbar^2/8m)\nabla^2\rho_b(\boldsymbol{r}_c) = H_b(\boldsymbol{r}_c) - V_b(\boldsymbol{r}_c)/2 \quad \text{(S2)}$$

$$= G_b(\boldsymbol{r}_c) + V_b(\boldsymbol{r}_c)/2 \quad \text{(S2')}$$

Interactions are classified by the signs of $\nabla^2\rho_b(\boldsymbol{r}_c)$ and $H_b(\boldsymbol{r}_c)$. Interactions in the region of $\nabla^2\rho_b(\boldsymbol{r}_c) < 0$ are called shared-shell (SS) interactions and they are closed-shell (CS) interactions for $\nabla^2\rho_b(\boldsymbol{r}_c) > 0$. $H_b(\boldsymbol{r}_c)$ must be negative when $\nabla^2\rho_b(\boldsymbol{r}_c) < 0$, since $H_b(\boldsymbol{r}_c)$ are larger than $(\hbar^2/8m)\nabla^2\rho_b(\boldsymbol{r}_c)$ by $V_b(\boldsymbol{r}_c)/2$ with negative $V_b(\boldsymbol{r}_c)$ at all BCPs (eqn (S2)). Consequently, $\nabla^2\rho_b(\boldsymbol{r}_c) < 0$ and $H_b(\boldsymbol{r}_c) < 0$ for the SS interactions. The CS interactions are especially called *pure* CS interactions for $H_b(\boldsymbol{r}_c) > 0$ and $\nabla^2\rho_b(\boldsymbol{r}_c) > 0$, since electrons at BCPs are depleted and destabilized under the conditions.[S1a] Electrons in the intermediate region between SS and *pure* CS, which belong to CS, are locally depleted but stabilized at BCPs, since $\nabla^2\rho_b(\boldsymbol{r}_c) > 0$ but

$H_b(\boldsymbol{r}_c) < 0$.[S1a] We call the interactions in this region *regular* CS,[S4,S5] when it is necessary to distinguish from *pure* CS. The role of $\nabla^2\rho_b(\boldsymbol{r}_c)$ in the classification can be replaced by $H_b(\boldsymbol{r}_c) - V_b(\boldsymbol{r}_c)/2$, since $(\hbar^2/8m)\nabla^2\rho_b(\boldsymbol{r}_c) = H_b(\boldsymbol{r}_c) - V_b(\boldsymbol{r}_c)/2$ (eqn (S2)).

We proposed QTAIM-DFA by plotting $H_b(\boldsymbol{r}_c)$ versus $H_b(\boldsymbol{r}_c) - V_b(\boldsymbol{r}_c)/2$ (= $(\hbar^2/8m)\nabla^2\rho_b(\boldsymbol{r}_c)$),[S4a] after the proposal of $H_b(\boldsymbol{r}_c)$ versus $\nabla^2\rho_b(\boldsymbol{r}_c)$.[S4b] Both axes in the plot of the former are given in energy unit, therefore, distances on the (x, y) (= $(H_b(\boldsymbol{r}_c) - V_b(\boldsymbol{r}_c)/2, H_b(\boldsymbol{r}_c))$ plane can be expressed in the energy unit, which provides an analytical development. QTAIM-DFA can incorporate the classification of interactions by the signs of $\nabla^2\rho_b(\boldsymbol{r}_c)$ and $H_b(\boldsymbol{r}_c)$. Scheme S1 summarizes the QTAIM-DFA treatment. Interactions of *pure* CS appear in the first quadrant, those of *regular* CS in the fourth quadrant and SS interactions do in the third quadrant. No interactions appear in the second one.

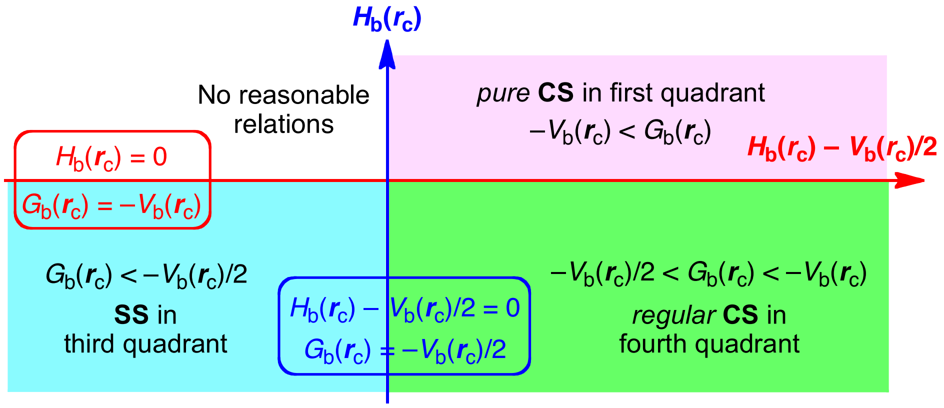

Scheme S1. QTAIM-DFA: Plot of $H_b(\boldsymbol{r}_c)$ versus $H_b(\boldsymbol{r}_c) - V_b(\boldsymbol{r}_c)/2$ for Weak to Strong Interactions

In our treatment, data for perturbed structures around fully optimized structures are also employed for the plots, together with the fully optimized ones (see Fig. S1).[S4–S6] We proposed the concept of the "dynamic nature of interaction" originated from the perturbed structures. The behavior of interactions at the fully optimized structures corresponds to "the static nature of interactions", whereas that containing perturbed structures exhibit the "dynamic nature of interaction" as explained below. The method to generate the perturbed structures is discussed later. Plots of $H_b(\boldsymbol{r}_c)$ versus $H_b(\boldsymbol{r}_c) - V_b(\boldsymbol{r}_c)/2$ are analyzed employing the polar coordinate (R, θ) representation with (θ_p, κ_p) parameters.[S4a,S5,S6] Fig. S1 explains the treatment. R in (R, θ) is defined by eqn (S3) and given in the energy unit. R corresponds to the energy for an interaction at BCP. The plots show a spiral stream, as a whole. θ in (R, θ) defined by eqn (S4), measured from the y-axis, controls the spiral stream of the plot. Each plot for an interaction shows a specific curve, which provides important information of the interaction (see Fig. S1). The curve is expressed by θ_p and κ_p. While θ_p, defined by eqn (S5) and measured from the y-direction, corresponds to the tangent line of a plot, where θ_p is calculated employing data of the perturbed structures with a fully-optimized structure and κ_p is the curvature of the plot (eqn (S6)). While (R, θ) correspond to the static nature, (θ_p, κ_p) represent the dynamic nature of interactions. We call (R, θ) and (θ_p, κ_p) QTAIM-DFA parameters, whereas $\rho_b(\boldsymbol{r}_c)$, $\nabla^2\rho_b(\boldsymbol{r}_c)$, $G_b(\boldsymbol{r}_c)$, $V_b(\boldsymbol{r}_c)$, $H_b(\boldsymbol{r}_c)$ and $H_b(\boldsymbol{r}_c) - V_b(\boldsymbol{r}_c)/2$ belong to QTAIM functions. $k_b(\boldsymbol{r}_c)$, defined by eqn (S7), is an QTAIM function but it will be treated as if it were an QTAIM-DFA parameter, if suitable.

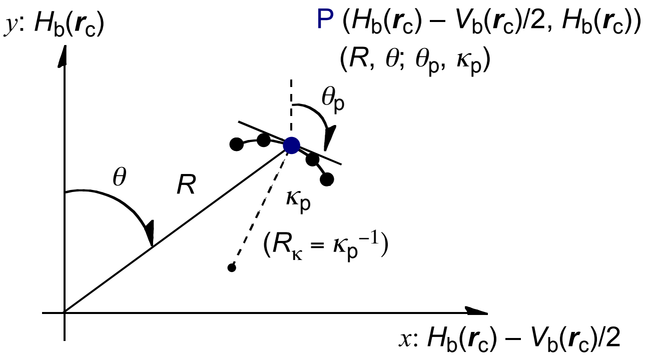

Figure S1. Polar (R, θ) coordinate representation of $H_b(\boldsymbol{r}_c)$ versus $H_b(\boldsymbol{r}_c) - V_b(\boldsymbol{r}_c)/2$, with ($\theta_p$, κ_p) parameters.

$$R = (x^2 + y^2)^{1/2} \quad \text{(S3)}$$
$$\theta = 90^\circ - \tan^{-1}(y/x) \quad \text{(S4)}$$
$$\theta_p = 90^\circ - \tan^{-1}(dy/dx) \quad \text{(S5)}$$
$$\kappa_p = |d^2y/dx^2| / [1 + (dy/dx)^2]^{3/2} \quad \text{(S6)}$$
$$k_b(\boldsymbol{r}_c) = V_b(\boldsymbol{r}_c)/G_b(\boldsymbol{r}_c) \quad \text{(S7)}$$

where $(x, y) = (H_b(\boldsymbol{r}_c) - V_b(\boldsymbol{r}_c)/2, H_b(\boldsymbol{r}_c))$

Criteria for Classification of Interactions: Behavior of Typical Interactions Elucidated by QTAIM-DFA

$H_b(\boldsymbol{r}_c)$ are plotted versus $H_b(\boldsymbol{r}_c) - V_b(\boldsymbol{r}_c)/2$ for typical interactions in vdW (van der Waals interactions), HB (hydrogen bonds), CT-MC (molecular complexes through charge transfer), X_3^- (trihalide ions), CT-TBP (trigonal bipyramidal adducts through charge-transfer), Cov-w (weak covalent bonds) and Cov-s (strong covalent bonds).[S4–S6] Rough criteria are obtained, after the analysis of the plots for the typical interactions according to eqns (S3)–(S7), by applying QTAIM-DFA. Scheme S2 shows the rough criteria, which are accomplished by the θ and θ_p values, together with the values of $k_b(\boldsymbol{r}_c)$. The criteria will be employed to discuss the nature of interactions in question, as a reference.

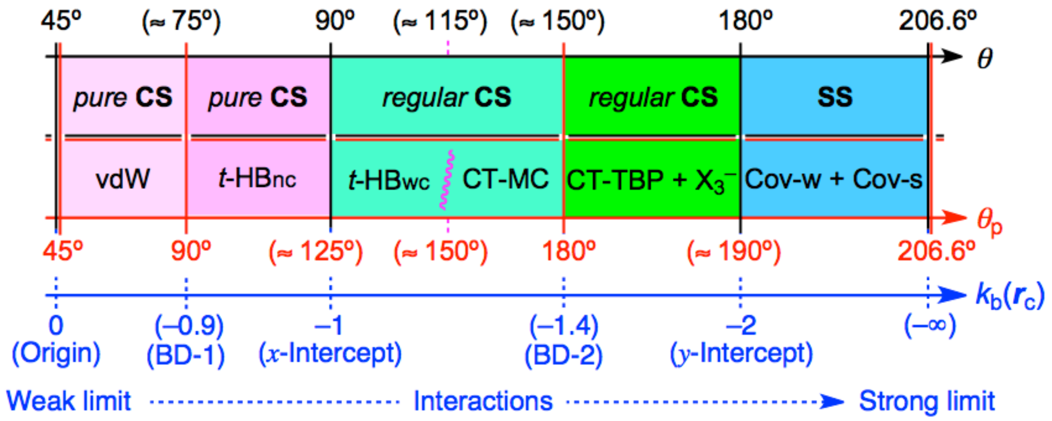

Scheme S2. Rough classification of interactions by θ and θ_p, together with $k_b(\boldsymbol{r}_c)$ (= $V_b(\boldsymbol{r}_c)/G_b(\boldsymbol{r}_c)$).

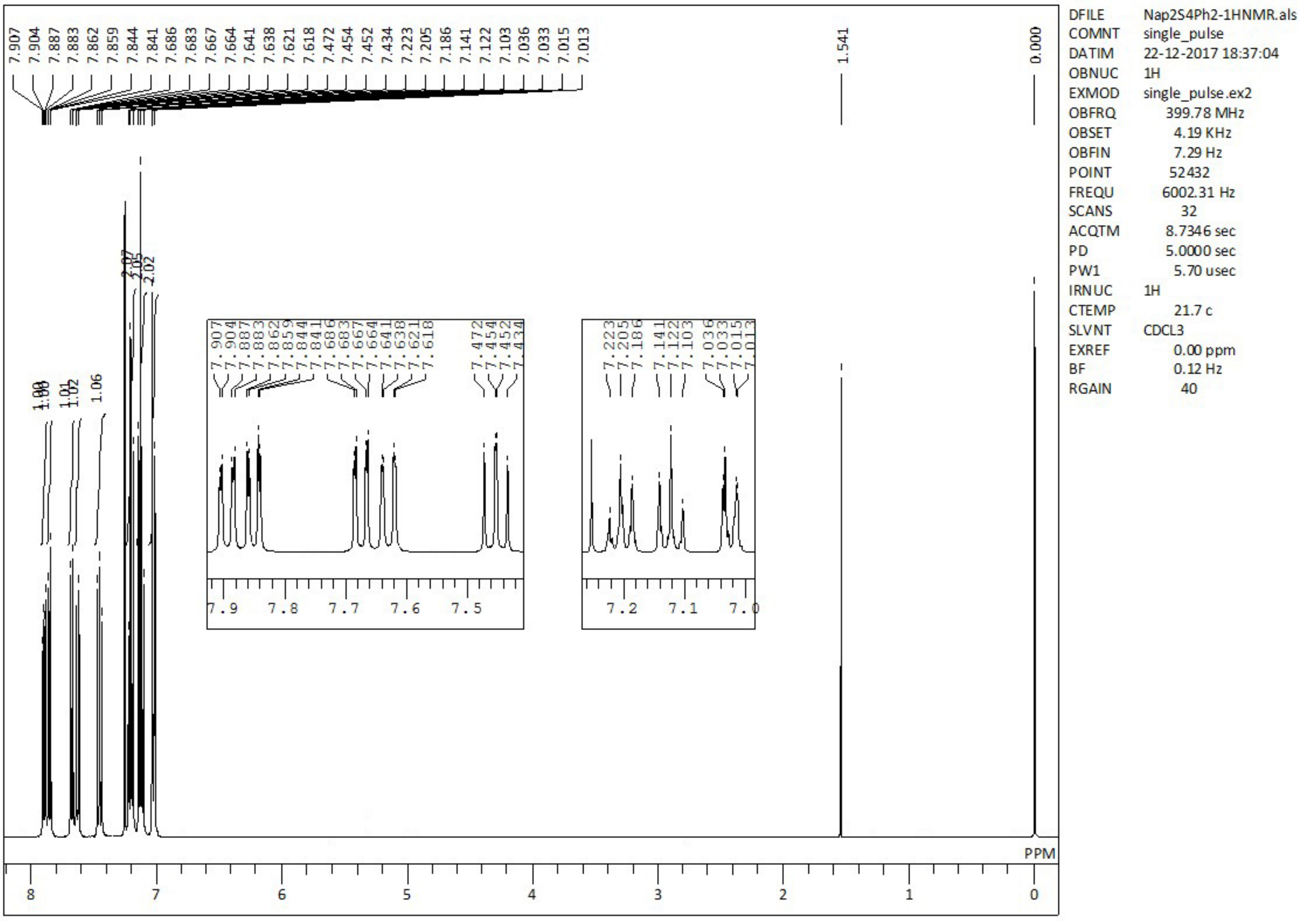

Fig. S2 ^{1}H NMR spectrum of **1** (S, S).

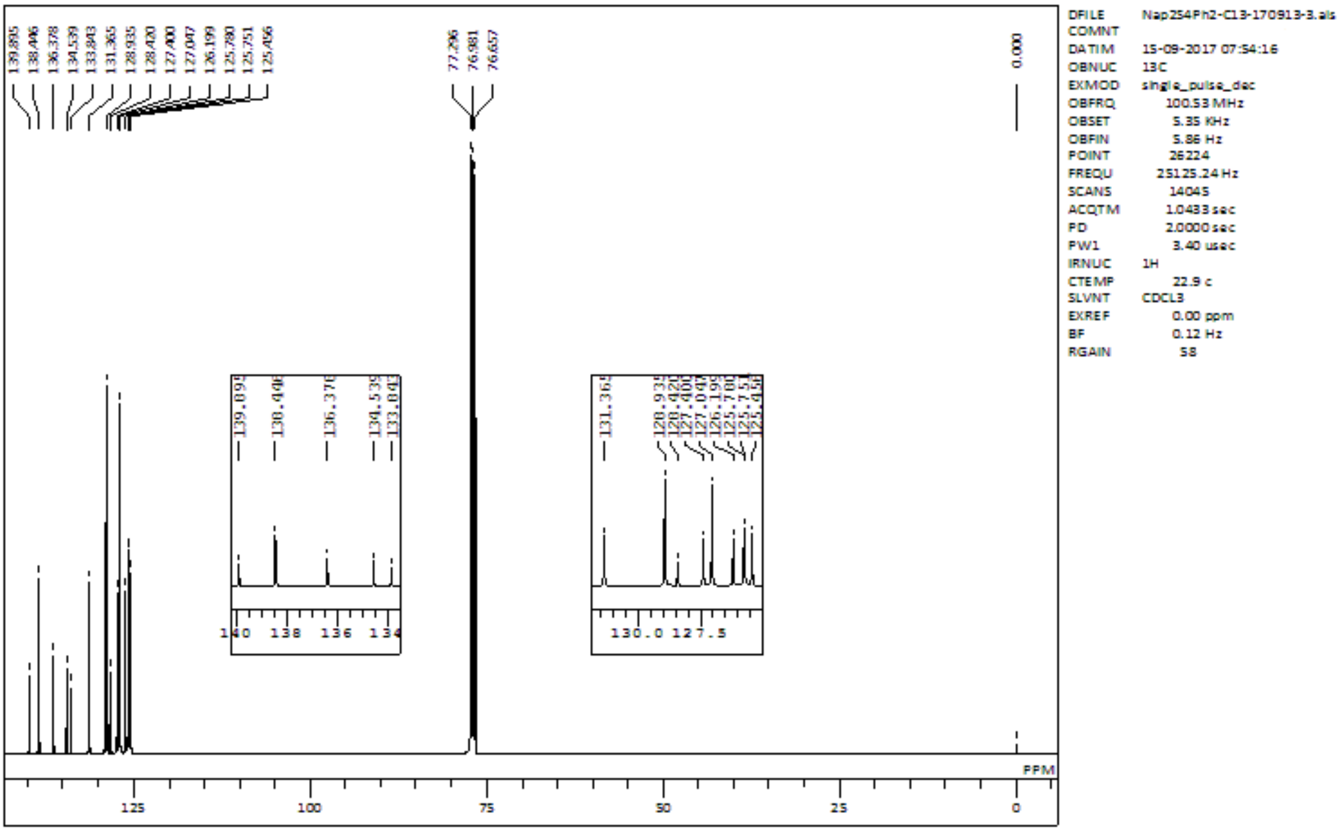

Fig. S3 ^{13}C NMR spectrum of **1** (S, S).

Table S1. Crystallographic data for **1** (S, S)

Empirical formula	$C_{32}H_{22}S_4$
Formula weight	534.73
Temperature (K)	100(2)
Crystal system	monoclinic
Space group	$P2_1/c$ (#14)
Unit cell dimensions	
a (Å)	10.4959(5)
b (Å)	23.8566(11)
c (Å)	10.3499(5)
β (°)	106.471(5)
Volume (Å^3)	2485.2(2)
Z	4
Theta range for data collection (°)	1.71–60.09
D_{calcd} (g cm^{-3})	1.429
F(000)	35199
Reflections observed [$I > 2\sigma(I)$]	38015
**Spherical refinement**	
R [F] and R [F^2]	0.0252, 0.0423
R_{all} [F] and R_{all} [F^2]	0.0283, 0.0424
R_w [F]and R_w [F^2]	0.0334, 0.0623
Goodness of fit	1.9487
Nref/Nv	85.2
**Multipole refinement**	
R [F] and R [F^2]	0.0165, 0.0205
R_{all} [F] and R_{all} [F^2]	0.0197, 0.0206
R_w [F]and R_w [F^2]	0.0235, 0.0412
Goodness of fit	1.3814
Nref/Nv	32.7
Residual density (eÅ^{-3})	-0.24 to 0.25

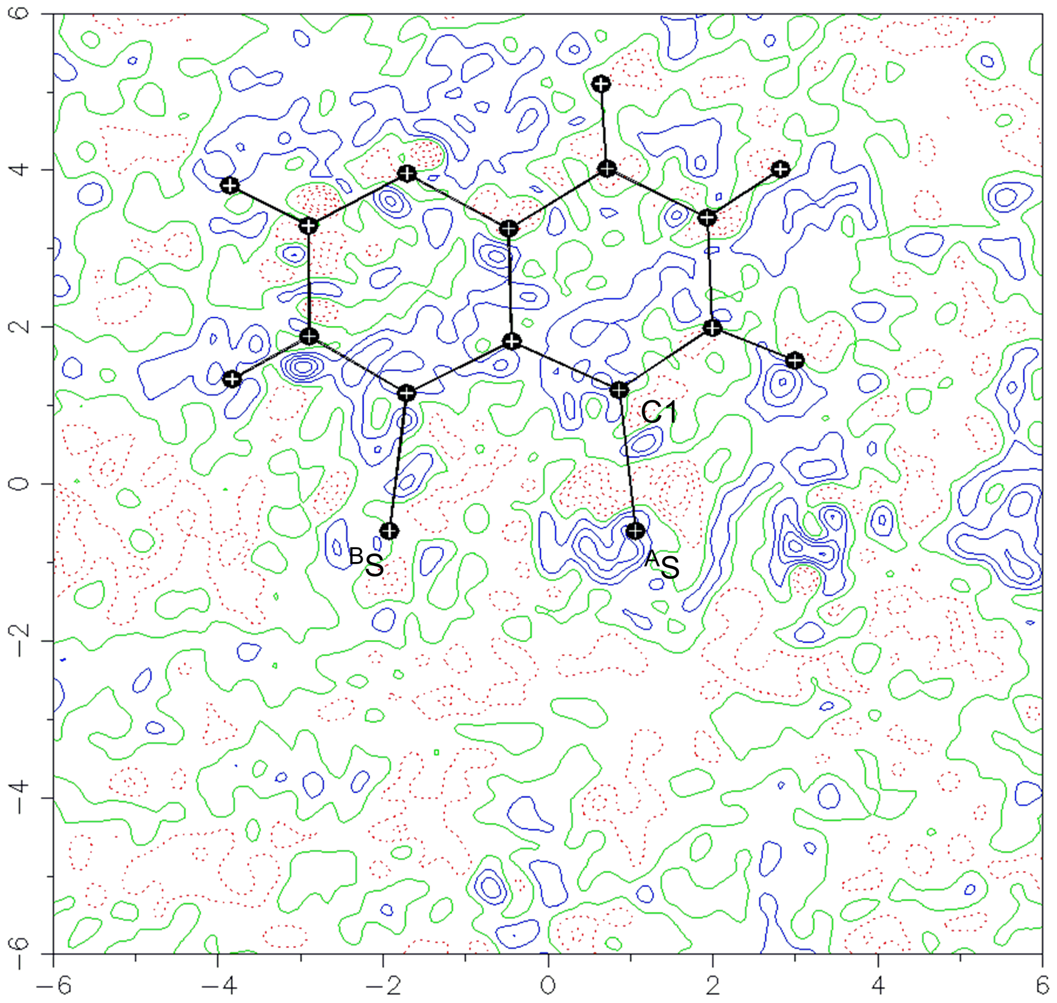

Fig. S4 Featureless residual density map of **1** (S, S) determined by final multipole refinement.

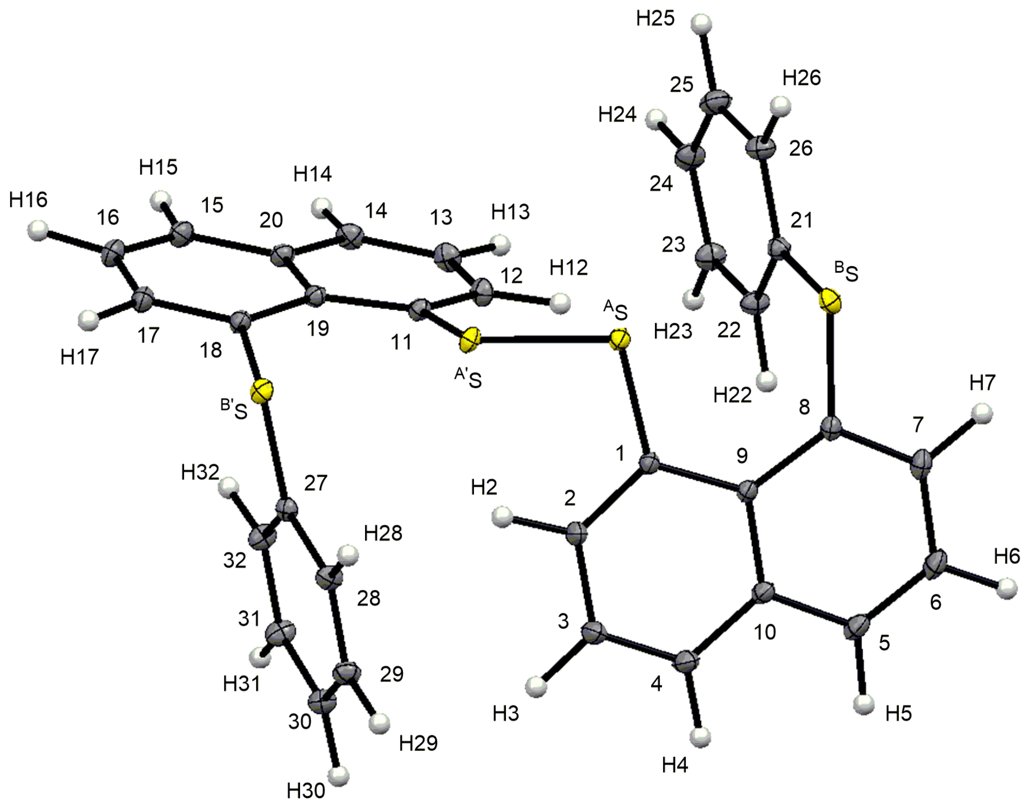

Fig. S5 Structure of **1** (S, S) determined by the high-resolution X-ray crystallographic analysis.

Table S2. Selected structural parameters observed for **1** (S, S) by the high resolution determined

	bond length (Å)		
^{A}S-^{B}S	2.9879(4)	$^{B'}$S-C27	1.7721(3)
^{A}S-$^{A'}$S	2.0559(5)	^{B}S-C8	1.7711(4)
$^{A'}$S-$^{B'}$S	2.9825(5)	^{B}S-C21	1.7720(3)
^{A}S-C1	1.8074(3)	^{A}S-H12	2.5043
$^{A'}$S-C11	1.8120(5)	$^{A'}$S-H2	2.4811
$^{B'}$S-C18	1.7726(2)		
	bond angles (º)		
^{B}S-^{A}S-$^{A'}$S	168.68(2)	C8-^{B}S-^{A}S	83.22(1)
^{A}S-$^{A'}$S-$^{B'}$S	165.70(2)	C18-$^{B'}$S-$^{A'}$S	82.28(1)
C1-^{A}S-$^{A'}$S	104.48(2)	C27-$^{B'}$S-$^{A'}$S	87.40(1)
C11-^{A}S-$^{A'}$S	105.54(2)	C9-C8-^{B}S	124.10(1)
C21-^{B}S-C8	102.99(2)	C9-C1-^{A}S	121.86(1)
C18-$^{B'}$S-C27	101.94(1)	C19-C11-$^{A'}$S	120.63(2)
C21-^{B}S-^{A}S	82.50(1)	C19-C18-$^{B'}$S	123.49(1)
	torsion angles (º)		
C1-^{A}S-$^{A'}$S-C11	–89.50(2)	^{A}S-C1-C9-C10	178.30(2)
C21-^{B}S-C8-C9	–80.98(2)	C26-C21-^{B}S-C8	174.60(1)
C27-$^{B'}$S-C18-C19	–70.22(2)	C28-C27-$^{B'}$S-C18	165.68(1)
^{A}S-$^{A'}$S-C11-C19	–175.35(2)	$^{B'}$S-$^{A'}$S-C11-C19	16.36(2)
$^{A'}$S-^{A}S-C1-C9	173.76(2)	^{B}S-^{A}S-C1-C9	1.77(2)
$^{A'}$S-C11-C19-C21	169.26(2)		

Table S3. R_{BP} and r_{SL} values for the ^{B}E ---^{A}E–^{A}E---^{B}E interactions in compounds **1**–**4**[a]

Species	$R_{SL}(^{A}E, ^{B}E)$ (Å)	$R_{SL}(^{A}E, ^{A}E)$ (Å)	$r_{BP}(^{A}E, ^{B}E)$ (Å)	$r_{BP}(^{A}E, ^{A}E)$ (Å)	$\Delta r_{BP}(^{A}E, ^{B}E)$[b] (Å)	$\Delta r_{BP}(^{A}E, ^{A}E)$[c] (Å)
**1** (S, S) (C_2)	2.9874	2.0730	3.0083	2.0742	0.0208	0.0012
**2** (S, Se) (C_2)	3.0673	2.0801	3.0842	2.0814	0.0170	0.0013
**3** (Se, S) (C_2)	3.0188	2.3512	3.0281	2.3524	0.0093	0.0012
**4** (Se, Se) (C_2)	3.0884	2.3642	3.0957	2.3655	0.0072	0.0013
**1** (S, S)$_{obsd}$ (C_1)	2.9879[d]	2.0559	3.0055[e,f]	2.0569[e]	0.0176	0.0010

[a] The 6-311+G(d) basis sets being employed for S, and Se with the 6-31G(d,p) basis sets for H and C. [b] $\Delta r_{BP}(^{A}E, ^{B}E) = r_{BP}(^{A}E, ^{B}E) - R_{SL}(^{A}E, ^{B}E)$. [c] $\Delta r_{BP}(^{A}E, ^{A}E) = r_{BP}(^{A}E, ^{A}E) - R_{SL}(^{A}E, ^{A}E)$. [d] Averaged value: $r_{obsd}(^{A}E, ^{B}E)$ = 2.9879(4) and 2.9825(5) Å. [e] r_{BP} value evaluated employing the observed structure. [f] Averaged value: $r_{BP:obsd}(^{A}E, ^{B}E)$ = 3.0037 and 3.0073 Å.

Table S4. Contributions from the CT interactions for the $n_p(^{B}E)\rightarrow\sigma^*(^{A}E–^{A}E)\leftarrow n_p(^{B}E)$ type (^{A}E, ^{B}E = S and Se) for **1**–**4**, evaluated with MP2/BSS-A[a,b]

Species ***n*** (^{A}E, ^{B}E)	NBO (i)[c]	NBO (j)[d]	$E(2)$ (kcal mol^{-1})	$E(j)$–$E(i)$ (au)	F(i,j) (au)
**1** (S, S)	n_p(S)	σ*(S–S)	6.58	0.38	0.045
**2** (S, Se)[e]	n_p(Se)	σ*(S–S)	7.36	0.37	0.046
**3** (Se, S)	n_p(S)	σ*(Se–Se)	10.81	0.34	0.054
**4** (Se, Se)[f]	n_p(Se)	σ*(Se–Se)	13.03	0.32	0.058

[a] BSS-A: the 6-311+G(d) basis set for S and Se with the 6-31G(d,p) basis sets for C and H. [b] Second order perturbation of Fock matrix (threshold being 0.50 kcal mol^{-1}). Only one side of the interaction is evaluated [c] Donor orbitals. [d] Acceptor orbitals. [e] E(2: n_s(Se)→σ*(S–S)) = 1.07 kcal mol^{-1}. [f] E(2: n_s(Se)→σ*(Se–Se)) = 1.09 kcal mol^{-1}.

References

S1 (a) *Atoms in Molecules. A Quantum Theory*: eds. R. F. W. Bader, Oxford University Press, Oxford, UK, 1990; (b) C. F. Matta, R. J. Boyd, *An Introduction to the Quantum Theory of Atoms in Molecules* in *The Quantum Theory of Atoms in Molecules: From Solid State to DNA and Drug Design*: eds. C. F. Matta, R. J. Boyd, WILEY-VCH, Weinheim, Germany, 2007, Chapter 1.

S2 (a) R. F. W. Bader, T. S. Slee. D. Cremer, E. Kraka, *J. Am. Chem. Soc.* **1983**, *105*, 5061–5068; (b) R. F. W. Bader, *Chem. Rev.* **1991**, *91*, 893–926; (c) R. F. W. Bader, *J. Phys. Chem. A* **1998**, *102*, 7314–7323; (d) F. Biegler-König, R. F. W. Bader, T. H. Tang, *J. Comput. Chem.* **1982**, *3*, 317–328; (e) R. F. W. Bader, *Acc. Chem. Res.* **1985**, *18*, 9–15; (f) T. H. Tang, R. F. W. Bader, P. MacDougall, *Inorg. Chem.* **1985**, *24*, 2047–2053; (g) F. Biegler-König, J. Schönbohm, D. Bayles, *J. Comput. Chem*. **2001**, *22*, 545–559; (h) F. Biegler-König, J. Schönbohm, *J. Comput. Chem.* **2002**, *23*, 1489–1494.

S3 W. Nakanishi, T. Nakamoto, S. Hayashi, T. Sasamori, N. Tokitoh, *Chem. Eur. J.* **2007**, *13*, 255–268.

S4 (a) W. Nakanishi, S. Hayashi, K. Narahara, *J. Phys. Chem. A* **2009**, *113*, 10050–10057; (*b*) W. Nakanishi, S. Hayashi, K. Narahara, *J. Phys. Chem. A* **2008**, *112*, 13593–13599.

S5 W. Nakanishi, S. Hayashi, *Curr. Org. Chem.* **2010**, *14*, 181–197.

S6 (a) W. Nakanishi, S. Hayashi, *J. Phys. Chem. A* **2010**, *114*, 7423–7430; (b) W. Nakanishi, S. Hayashi, K. Matsuiwa, M. Kitamoto, *Bull. Chem. Soc. Jpn* **2012**, *85*, 1293–1305.

Optimized structures given by Cartesian coordinates

Calculations are performed using the Gaussian 09 program package. Compound **1** (S, S) was optimized with the 6-311+G(d) basis set for S and the 6-31G(d,p) basis sets for C and H. The basis set system is called A (BSS-A), in this paper. The Møller-Plesset second order energy correlation (MP2) level was applied to the calculations. Structural parameters optimized with MP2/BSS-A ($r(^{A}S, ^{A'}S)$ = 2.0730 Å and $r(^{A}S, ^{B}S)$ = 2.9874 Å) were very close to the observed values ($r_{obsd}(^{A}S, ^{A'}S)$ = 2.0559(5) Å and $r_{obsd:av}(^{A}S, ^{B}S)$ = 2.9852 Å), respectively. Compounds **1**–**4** were similarly optimized with MP2/BSS-A, where the 6-311+G(d) basis sets were applied for S and/or Se with the 6-31G(d,p) basis sets for C and H.

MP2/BSS-A
Compound **1** (S, S)
Symmetry C_2
energy MP2 = -2819.4713724 au
Standard orientation

6	0	0.533344	4.073292	-0.686251
6	0	1.241118	2.971401	-0.097041
6	0	2.266481	3.318747	0.861217
6	0	2.697876	4.664559	0.993436
6	0	2.076794	5.682904	0.305739
6	0	0.977293	5.380432	-0.518862
6	0	0.947172	1.574177	-0.274724
6	0	1.586393	0.629582	0.521369
6	0	2.532624	0.999090	1.494434
6	0	2.915129	2.314675	1.620970
1	0	3.504291	4.878270	1.686829
1	0	2.408675	6.708494	0.412781
1	0	0.410388	6.179663	-0.981396
1	0	1.364119	-0.420368	0.389258
1	0	2.998275	0.222605	2.087909
1	0	3.678990	2.610540	2.331765
16	0	-0.947172	3.897286	-1.652181
16	0	-0.104725	1.031193	-1.629258
6	0	-0.533344	-4.073292	-0.686251
6	0	-1.241118	-2.971401	-0.097041
6	0	-2.266481	-3.318747	0.861217
6	0	-2.697876	-4.664559	0.993436
6	0	-2.076794	-5.682904	0.305739
6	0	-0.977293	-5.380432	-0.518862
6	0	-0.947172	-1.574177	-0.274724
6	0	-1.586393	-0.629582	0.521369
6	0	-2.532624	-0.999090	1.494434
6	0	-2.915129	-2.314675	1.620970
1	0	-3.504291	-4.878270	1.686829
1	0	-2.408675	-6.708494	0.412781
1	0	-0.410388	-6.179663	-0.981396
1	0	-1.364119	0.420368	0.389258
1	0	-2.998275	-0.222605	2.087909
1	0	-3.678990	-2.610540	2.331765

16	0	0.947172	-3.897286	-1.652181
16	0	0.104725	-1.031193	-1.629258
6	0	-2.061183	3.250876	-0.427930
6	0	-1.894475	3.467845	0.947141
6	0	-2.835343	2.958592	1.846757
6	0	-3.953630	2.257697	1.386033
6	0	-4.118043	2.044307	0.013889
6	0	-3.170737	2.526098	-0.890508
1	0	-1.040942	4.027269	1.310931
1	0	-2.700316	3.129263	2.908634
1	0	-4.689524	1.883659	2.087889
1	0	-4.972609	1.488718	-0.353637
1	0	-3.280104	2.332075	-1.951941
6	0	2.061183	-3.250876	-0.427930
6	0	3.170737	-2.526098	-0.890508
6	0	4.118043	-2.044307	0.013889
6	0	3.953630	-2.257697	1.386033
6	0	2.835343	-2.958592	1.846757
6	0	1.894475	-3.467845	0.947141
1	0	3.280104	-2.332075	-1.951941
1	0	4.972609	-1.488718	-0.353637
1	0	4.689524	-1.883659	2.087889
1	0	2.700316	-3.129263	2.908634
1	0	1.040942	-4.027269	1.310931

MP2/BSS-A
Compound **2** (S, Se)
Symmetry C_2
energy MP2 = -6824.2493241 au
Standard orientation

6	0	-1.075618	4.011508	-0.660104
6	0	-0.018968	3.279407	-0.023908
6	0	0.826240	4.042937	0.867788
6	0	0.759469	5.460500	0.879569
6	0	-0.186599	6.135739	0.140810
6	0	-1.125203	5.400175	-0.607779
6	0	0.203217	1.859661	-0.093281
6	0	1.125203	1.266528	0.763499
6	0	1.882862	2.022167	1.677251
6	0	1.786383	3.394781	1.683460
1	0	1.445164	6.000652	1.523703
1	0	-0.235820	7.217853	0.157756
1	0	-1.949363	5.911848	-1.090271
1	0	1.280352	0.197011	0.723744
1	0	2.587741	1.508884	2.318987
1	0	2.402655	3.997197	2.342035
34	0	-2.562579	3.192345	-1.563664
16	0	-0.553893	0.880293	-1.396679
6	0	1.075618	-4.011508	-0.660104

6	0	0.018968	-3.279407	-0.023908
6	0	-0.826240	-4.042937	0.867788
6	0	-0.759469	-5.460500	0.879569
6	0	0.186599	-6.135739	0.140810
6	0	1.125203	-5.400175	-0.607779
6	0	-0.203217	-1.859661	-0.093281
6	0	-1.125203	-1.266528	0.763499
6	0	-1.882862	-2.022167	1.677251
6	0	-1.786383	-3.394781	1.683460
1	0	-1.445164	-6.000652	1.523703
1	0	0.235820	-7.217853	0.157756
1	0	1.949363	-5.911848	-1.090271
1	0	-1.280352	-0.197011	0.723744
1	0	-2.587741	-1.508884	2.318987
1	0	-2.402655	-3.997197	2.342035
34	0	2.562579	-3.192345	-1.563664
16	0	0.553893	-0.880293	-1.396679
6	0	-3.232948	2.178179	-0.073567
6	0	-3.054788	2.602356	1.249923
6	0	-3.580625	1.835359	2.293502
6	0	-4.302506	0.668641	2.022823
6	0	-4.481107	0.253924	0.699270
6	0	-3.935152	0.996503	-0.350580
1	0	-2.513913	3.517095	1.462362
1	0	-3.438921	2.161809	3.317348
1	0	-4.725598	0.090537	2.835967
1	0	-5.025558	-0.657473	0.481699
1	0	-4.038924	0.652856	-1.373928
6	0	3.232948	-2.178179	-0.073567
6	0	3.935152	-0.996503	-0.350580
6	0	4.481107	-0.253924	0.699270
6	0	4.302506	-0.668641	2.022823
6	0	3.580625	-1.835359	2.293502
6	0	3.054788	-2.602356	1.249923
1	0	4.038924	-0.652856	-1.373928
1	0	5.025558	0.657473	0.481699
1	0	4.725598	-0.090537	2.835967
1	0	3.438921	-2.161809	3.317348
1	0	2.513913	-3.517095	1.462362

MP2/BSS-A
Compound **3** (Se, S)
Symmetry C_2
energy MP2 = -6824.2797335 au
Standard orientation

6	0	-1.099583	4.135030	-0.548371
6	0	-0.010995	3.381246	0.000076
6	0	0.862961	4.088958	0.907350
6	0	0.784603	5.502793	1.009164

6	0	-0.195359	6.211918	0.349084
6	0	-1.155501	5.516609	-0.411346
6	0	0.207399	1.973624	-0.168460
6	0	1.155501	1.321432	0.610442
6	0	1.947771	2.019771	1.542796
6	0	1.854723	3.390576	1.639742
1	0	1.488752	6.011013	1.659476
1	0	-0.250434	7.290422	0.434557
1	0	-1.999029	6.046557	-0.837965
1	0	1.310686	0.256366	0.494448
1	0	2.676494	1.467027	2.123102
1	0	2.497705	3.949275	2.311337
16	0	-2.472203	3.369334	-1.372782
34	0	-0.631873	0.991339	-1.640476
6	0	1.099583	-4.135030	-0.548371
6	0	0.010995	-3.381246	0.000076
6	0	-0.862961	-4.088958	0.907350
6	0	-0.784603	-5.502793	1.009164
6	0	0.195359	-6.211918	0.349084
6	0	1.155501	-5.516609	-0.411346
6	0	-0.207399	-1.973624	-0.168460
6	0	-1.155501	-1.321432	0.610442
6	0	-1.947771	-2.019771	1.542796
6	0	-1.854723	-3.390576	1.639742
1	0	-1.488752	-6.011013	1.659476
1	0	0.250434	-7.290422	0.434557
1	0	1.999029	-6.046557	-0.837965
1	0	-1.310686	-0.256366	0.494448
1	0	-2.676494	-1.467027	2.123102
1	0	-2.497705	-3.949275	2.311337
16	0	2.472203	-3.369334	-1.372782
34	0	0.631873	-0.991339	-1.640476
6	0	-3.145275	2.414287	-0.029543
6	0	-2.994152	2.785668	1.313797
6	0	-3.562962	1.996910	2.316875
6	0	-4.308741	0.860273	1.990693
6	0	-4.471834	0.502177	0.648806
6	0	-3.884148	1.267998	-0.359716
1	0	-2.429347	3.673030	1.573332
1	0	-3.435903	2.284204	3.354123
1	0	-4.761975	0.263448	2.773374
1	0	-5.040806	-0.381299	0.384313
1	0	-3.979704	0.969983	-1.397726
6	0	3.145275	-2.414287	-0.029543
6	0	3.884148	-1.267998	-0.359716
6	0	4.471834	-0.502177	0.648806
6	0	4.308741	-0.860273	1.990693
6	0	3.562962	-1.996910	2.316875
6	0	2.994152	-2.785668	1.313797
1	0	3.979704	-0.969983	-1.397726

1	0	5.040806	0.381299	0.384313
1	0	4.761975	-0.263448	2.773374
1	0	3.435903	-2.284204	3.354123
1	0	2.429347	-3.673030	1.573332

MP2/BSS-A
Compound **4** (Se, Se)
Symmetry C_2
energy MP2 = -10829.0581797 au
Standard orientation

6	0	-1.445571	4.069453	-0.474751
6	0	-0.320215	3.396884	0.101974
6	0	0.491286	4.184884	1.003942
6	0	0.320215	5.592535	1.069495
6	0	-0.695864	6.220923	0.382922
6	0	-1.600520	5.446430	-0.369814
6	0	-0.004836	2.002402	-0.025930
6	0	0.973512	1.437823	0.784475
6	0	1.702275	2.210724	1.709577
6	0	1.516106	3.573913	1.768268
1	0	0.981385	6.160403	1.715599
1	0	-0.823270	7.295120	0.442794
1	0	-2.474325	5.911714	-0.810259
1	0	1.202175	0.383238	0.699704
1	0	2.456217	1.723085	2.315328
1	0	2.108624	4.191991	2.434316
34	0	-2.887379	3.137447	-1.335335
34	0	-0.744058	0.918558	-1.480358
6	0	1.445571	-4.069453	-0.474751
6	0	0.320215	-3.396884	0.101974
6	0	-0.491286	-4.184884	1.003942
6	0	-0.320215	-5.592535	1.069495
6	0	0.695864	-6.220923	0.382922
6	0	1.600520	-5.446430	-0.369814
6	0	0.004836	-2.002402	-0.025930
6	0	-0.973512	-1.437823	0.784475
6	0	-1.702275	-2.210724	1.709577
6	0	-1.516106	-3.573913	1.768268
1	0	-0.981385	-6.160403	1.715599
1	0	0.823270	-7.295120	0.442794
1	0	2.474325	-5.911714	-0.810259
1	0	-1.202175	-0.383238	0.699704
1	0	-2.456217	-1.723085	2.315328
1	0	-2.108624	-4.191991	2.434316
34	0	2.887379	-3.137447	-1.335335
34	0	0.744058	-0.918558	-1.480358
6	0	-3.424603	2.081725	0.183166
6	0	-3.247428	2.537540	1.495918
6	0	-3.656333	1.731693	2.561968

6	0	-4.261652	0.493513	2.324524
6	0	-4.446966	0.051516	1.010761
6	0	-4.015848	0.834784	-0.063081
1	0	-2.793378	3.503553	1.683050
1	0	-3.512824	2.080498	3.578083
1	0	-4.592312	-0.117769	3.156012
1	0	-4.906937	-0.910678	0.818114
1	0	-4.123634	0.473612	-1.079674
6	0	3.424603	-2.081725	0.183166
6	0	4.015848	-0.834784	-0.063081
6	0	4.446966	-0.051516	1.010761
6	0	4.261652	-0.493513	2.324524
6	0	3.656333	-1.731693	2.561968
6	0	3.247428	-2.537540	1.495918
1	0	4.123634	-0.473612	-1.079674
1	0	4.906937	0.910678	0.818114
1	0	4.592312	0.117769	3.156012
1	0	3.512824	-2.080498	3.578083
1	0	2.793378	-3.503553	1.683050
